# Tunable Hierarchically Porous Gadolinium‐Based Metal‐Organic Frameworks for Bacteria‐Targeting Magnetic Resonance Imaging and In Situ Anti‐Bacterial Therapy

**DOI:** 10.1002/advs.202415209

**Published:** 2025-02-20

**Authors:** Youyi Yu, Tianming Cui, Chang Liu, Weitao Yang, Bingbo Zhang

**Affiliations:** ^1^ Department of Radiology Tongji Hospital Shanghai Frontiers Science Center of Nanocatalytic Medicine the Institute for Biomedical Engineering & Nano Science School of Medicine Tongji University Shanghai 200065 China; ^2^ Shanghai Research Institute for Intelligent Autonomous Systems Tongji University Shanghai 200065 China

**Keywords:** bacteria targeting, hierarchical porous structure, magnetic resonance imaging, nanoprobes, theranostics

## Abstract

Currently, there are no non‐invasive tools to accurately diagnose deep surgical site bacterial infections before they cause significant anatomical damage in the clinic. An urgent need exists for bacteria‐targeting bifunctional probes for the detection of deep bacterial infections and precise in situ treatment. Herein, the bacteria‐targeting 1‐borono‐3,5‐benzenedicarboxylic acid (BBDC) ligand and paramagnetic Gd^3 + ^into one single metal‐organic frameworks (MOFs) are integrated, synergistically realizing bacteria‐specific magnetic resonance imaging (MRI) diagnosis and MRI‐guided antibacterial treatment. Molecular simulations and nitrogen adsorption‐desorption experiments demonstrate that a hierarchical porous structure can be constructed by tuning the Gd^3 +^ /BBDC ratio, which endows the Gd‐BBDC1.25 MOFs with an impressive longitudinal proton relaxivity of 15.81 mM^−1^ s^−1^. In particular, the bacteria‐targeting boronic acid group in BBDC remained intact during the MOF synthesis, ensuring that Gd‐BBDC1.25 MOFs have a unique combination of high sensitivity and specificity for bacteria. Through an in situ reduction reaction, silver nanoparticles （Ag NPs）‐modified Gd‐BBDC1.25 MOFs to form Ag@Gd‐BBDC1.25, an interfacial Schottky heterojunction nanozyme, which enhances their peroxidase (POD)‐catalyze activity. Furthermore, it is demonstrated that the bacteria‐targeting Ag@Gd‐BBDC1.25 bifunctional probe can image as few as 10^5^ colony‐forming units (cfu) in vivo and effectively eradicate the bacteria in situ.

## Graphical abstract

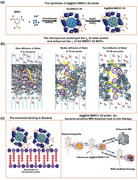

## 1 Introduction

Bacterial infections are one of the most severe health risks worldwide, and accurate diagnosis and effective treatment remain a significant challenge in clinical settings.^[^ ^1^, ^2^, ^3^ ^]^ To address this challenge, the development of accurate diagnostic methods and synergistic therapeutic techniques is highly needed.^[^ ^4^, ^5^ ^]^ Currently, clinical imaging modalities, including X‐ray computed tomography (CT),^[^ ^6^ ^]^ magnetic resonance imaging (MRI),^[^ ^7^, ^8^, ^9^ ^]^ and positron emission tomography (PET)^[^ ^10^, ^11^ ^]^ have provided valuable anatomical information but are only helpful in diagnosing delayed and late‐stage bacterial infections. Also, these imaging modalities cannot differentiate bacterial infections from sterile inflammation or tumor in situ. To improve sensitivity, specificity, and earlier detection of bacteria themselves, a molecular imaging strategy is really needed. Bacteria‐targeted optical molecular imaging (FLI) has attracted attention due to high sensitivity, real‐time imaging capability, and ease of use.^[^ ^12^, ^13^, ^14^, ^15^ ^]^ However, the limited penetration depth of FLI limits its application in imaging deep‐seated bacterial infections. CT and nuclear imaging have their limitations due to radioactivity.

Currently, bacteria‐targeted MRI probes are capturing increasing attention due to high penetration depth, high soft tissue resolution, and no radiation. Hu's group developed gadolinium (Gd)‐based bacteria‐targeted MRI probe (r_1_ = 4.05 mM^−1^ s^−1^) by conjugating an aminoglycoside antibiotic with Gd‐DOTA. The probe exhibited enhanced MRI signals than Gd‐DOTA‐treated bacteria in vitro and in vivo.^[^ ^16^ ^]^ Xu et al. reported an MRI probe (r_1_ = 11.48 mM^−1^ s^−1^) by conjugating Gd_2_O_3_ with maltodextrin for specific diagnosis of bacterial infection in vivo. The resulting probe was able to target bacteria cells and distinguish bacterial infections from tumors.^[^ ^17^ ^]^ A self‐assembling peptide (FFYEGK) and vancomycin (Van) antibiotic co‐modified Gd‐based MRI nanoaggregate probe was synthesized by Song's group; the as‐prepared probe carried Van molecules, which made it good for bacteria‐specific targeting, and the peptide in the probe can enhance the longitudinal relaxivity rate after self‐assembly due to the π–π stacking.^[^ ^18^ ^]^ However, those MRI probes have involved complex and multi‐step organic syntheses to conjugate various bacterial recognition moieties to Gd‐based contrast agents. Moreover, although these strategies have improved the specificity of probes towards bacteria, their sensitivity remains a limiting factor for their wider applications.

**Scheme 1:**
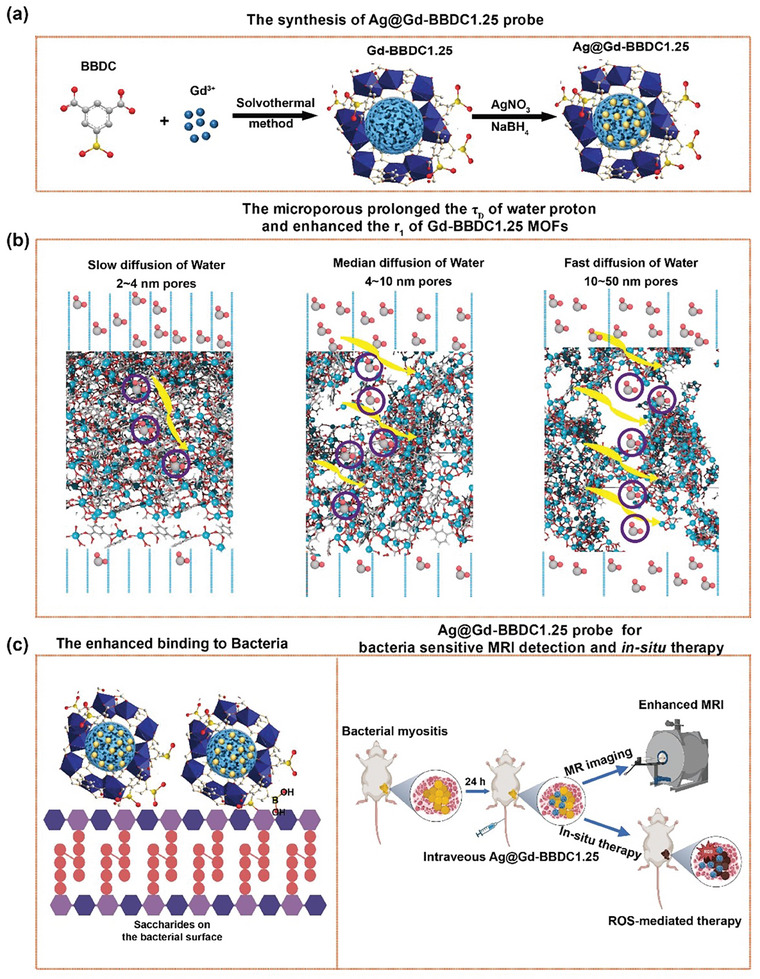
Schematic illustration of Ag@Gd‐BBDC1.25 nanoprobe for in vivo MRI and eradication of bacteria. a) The preparation process of Ag@Gd‐BBDC1.25 nanoprobe. b) Schematic illustrating the underlying mechanism of enhanced relaxivity. c) Bacteria‐targeted hierarchical porous Ag@Gd‐BBDC1.25 bifunctional nanoprobe realized sensitive and specific MRI detection of bacteria and ROS‐mediated in situ therapy.

Metal‐organic frameworks (MOFs) (Table S1, Supporting Information) have been investigated as a highly promising class of materials for developing MRI contrast agents due to their huge design variety and high density of metal ions.^[^ ^19^, ^20^, ^21^, ^22^, ^23^, ^24^ ^]^ However, for Gd‐based MOFs, carboxylate ligands such as benzene‐1,4‐dicarboxylate and benzene‐1,3,5‐tricarboxylate and N‐(4‐carboxybenzyl)‐(3,5‐dicarboxyl)‐pyridinium bromide used for Gd‐based MOFs tend to form clusters with metal ions, thus somehow preventing the water molecular access to Gd(III) paramagnetic center. Geometric confinement strategy, which confines Gd(III) paramagnetic center within the channels of porous materials, has become a very appealing strategy to improve the relaxivity of MRI contrast agents, which can not only restrict the rotation of the molecules but also trap the diffusion of the water molecules within the nanopores,^[^ ^25^ ^]^ and thus can significantly improve the relaxivity of MRI probes by increasing both τ_R_ and the water diffusion correlation time (τ_D_).^[^ ^25^ ^]^ Therefore, MOFs with adjustable pores, water accessibility, and bacteria‐targeting capabilities are essential for specific and sensitive MRI detection of deep‐seated bacterial infections. In this study, we report an effective strategy for integrating the bacteria‐targeting 1‐borono‐3,5‐benzenedicarboxylic acid (BBDC) ligand with paramagnetic Gd^3 +^ to construct a series of hierarchically porous Gd‐BBDC MOFs via a convenient one‐pot solvothermal method. Molecular simulations and nitrogen adsorption‐desorption experiments demonstrate that the pore size can be tuned by varying the reaction ratio of BBDC/Gd^3 +^. Ultimately, The Gd‐BBDC1.25 MOFs prepared at a concentration of 1.25 mmol L^−1^ BBDC, which has the largest percentage of 2–4 nm pore size and exhibits a high longitudinal proton relaxation (r_1_ of 15.81 mM^−1^ s^−1^). We further modified Gd‐BBDC1.25 MOFs with silver nanoparticles (Ag NPs) to form Ag@Gd‐BBDC1.25 bifunctional probe via an in situ reduction reaction. The results show that Ag@Gd‐BBDC1.25 nanoprobe can specifically accumulate at the bacterial infection site with enhanced MRI signals for accurate bacterial diagnosis and simultaneously enhanced peroxidase (POD)‐catalyzed anti‐bacteria treatment in vivo (**Scheme** **1**).

## 2 Results and Discussion

### 2.1 Preparation and Characterization of Gd‐BBDC MOFs

**Figure 1:**
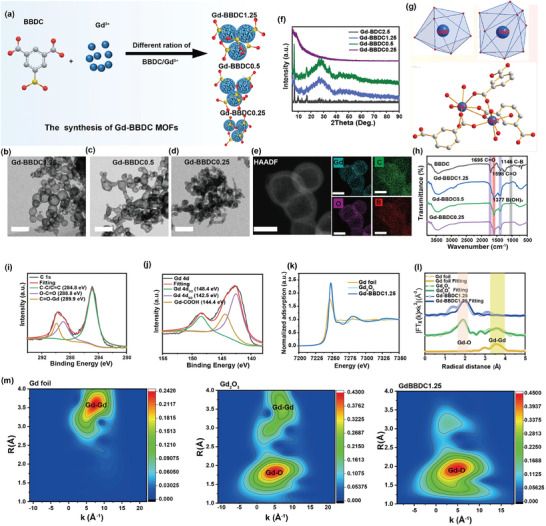
Characterization of Gd‐BBDC MOFs. a) Scheme of the fabrication of Gd‐BBDC MOFs. b–d) TEM images of Gd‐BBDC1.25, Gd‐BBDC0.5 and Gd‐BBDC0.25 MOFs, scale bar: 200 nm. e) HAADF‐STEM images of Gd‐BBDC1.25 and corresponding elemental mapping, scale bar: 50 nm. f) Powder XRD patterns of Gd‐BBDC1.25, Gd‐BBDC0.5, Gd‐BBDC0.25 and Gd‐BDC2.5. g) Coordination environments of the two types of independent Gd^3 + ^ions in the building unit of Gd_2_(BDC)_3_(H_2_O)_2_ MOF and coordination geometries of Gd1 and Gd2 ions. The building unit of Gd_2_(BDC)_3_(H_2_O)_2_ MOF was drawn by Diamond software. h) FTIR spectra of BBDC ligand and Gd‐BBDC MOFs. High‐resolution XPS spectra of i) C1s and j) Gd 3d. k) Gd L_3_‐edge XANES spectra of Gd‐BBDC1.25. l) Fourier‐transforms of K^3^‐weight Gd L_3_‐edge EXAFS spectra and corresponding fitting results. m) Wavelet transforms for K^3^‐weight Gd L_3_‐edge EXAFS signals.

We synthesized hierarchically porous Gd‐BBDC MOFs via a one‐step solvothermal method, using BBDC as the organic linker and GdCl_3_^.^6H_2_O as the metal node. By tuning the BBDC‐to‐Gd^3 + ^molar ratio, we achieved extensive optimization of the MOFs' size, morphology, and pore distribution (**Figure** **1**a). The size of Gd‐BBDC MOFs reduced from 90 to 40 nm by varying the BBDC concentration from 1.25 to 0.25 mmol L^−1^ (Figure 1b–d; Figure S1, Supporting Information). Notably, when the BBDC concentration reached up to 1.25 mmol L^−1^, the best size uniformity was obtained, whereas a higher concentration of 2.5 mmol L^−1^ led to a non‐uniform size distribution in the Gd‐BBDC2.5 MOFs (Figure S2, Supporting Information). High‐angle annular dark field scanning transmission electron microscopy (HAADF‐STEM) and energy dispersive X‐ray spectroscopy (EDS) mapping confirmed the homogeneous elemental distribution of carbon (C), oxygen (O), boron (B), and gadolinium (Gd) within the Gd‐BBDC1.25 MOFs (Figure 1e).

To investigate whether boric acid groups of BBDC participate in chelation with Gd^3 + ^during the synthesis of Gd‐BBDC MOFs, we have synthesized Gd‐BDC2.5 MOFs under identical conditions but using the non‐boric acid‐containing ligand 1,3‐benzenedicarboxylic acid (BDC) instead of BBDC.^[^ ^26^ ^]^ TEM showed that the Gd‐BDC2.5 MOFs had a similar morphology and size to the Gd‐BBDC1.25 MOFs (Figure S3, Supporting Information). The powder X‐ray diffraction (XRD) patterns (Figure 1f) revealed that the main peaks of the synthesized Gd‐BBDC1.25 and Gd‐BBDC0.5 MOFs were well matched with the phase‐pure Gd_2_(BDC)_3_(H_2_O)_2_ MOFs, which is consistent with literature reports.^[^ ^26^, ^27^ ^]^ This structural consistency indicates that Gd‐BBDC MOFs retain chelation chemistry akin to Gd_2_(BDC)_3_(H_2_O)_2_, suggesting that the boronic acid group does not coordinate with Gd^3 +^ and remains available as a functional site for bacterial targeting. Because Gd‐BBDC MOFs and Gd_2_(BDC)_3_(H_2_O)_2_ were isostructural and the formation of single‐crystal Gd‐BBDC MOFs was difficult, the single‐crystal XRD pattern of Gd_2_(BDC)_3_(H_2_O)_2_ was selected to reveal the chemical coordination environment of gadolinium ions in Gd‐BBDC MOFs. The fundamental building block of Gd_2_(BDC)_3_(H_2_O)_2_ is composed of three deprotonated BDC ligands, two distinct gadolinium (Gd) atoms, and two water (H_2_O) molecules (Figure 1g). The Gd1 atoms are seven‐coordinate, forming a pentagonal bipyramidal geometry, whereas the Gd2 atoms are coordinated by nine oxygen atoms derived from three BDC ligands and one water molecule.

Fourier transform infrared (FTIR) spectra of BBDC, Gd‐BBDC1.25, Gd‐BBDC0.5, and Gd‐BBDC0.25 were recorded respectively (Figure 1h). The disappearance of the peak at 1695 cm^−1^, corresponding to the carbonyl (νC═O) stretching vibration of the ligands in the Gd‐BBDC MOFs, confirmed the coordination of carboxylate groups with Gd^3 +^ ions. However, the presence of a B‐O absorption peak at 1377 cm^−1^ in both BBDC and Gd‐BBDC MOFs indicated that the boric acid sites remained free,^[^ ^26^ ^]^ which is crucial for their bacterial targeting capability. The full‐survey X‐ray photoelectron spectroscopy (XPS) spectra confirmed the elemental composition of C, O, B, and Gd in the Gd‐BBDC MOFs (Figure S4, Supporting Information). High‐resolution XPS analysis identified peaks at 284.8, 288.8, and 289.9 eV in the C 1s spectrum, corresponding to (C─C/C═C), O─C═O, and C═O─Gd bonds, respectively (Figure 1i).^[^ ^28^ ^]^ Additionally, the O 1s XPS peaks at 531.8 and 532.9 eV were attributed to C═O and C─OH groups, respectively (Figure S5, Supporting Information).^[^ ^29^ ^]^ The characteristic peaks for Gd 3d_3/2_, Gd 3d_5/2_, Gd 4d_3/2_, and Gd 4d_5/2_ in Gd‐BBDC1.25 (Figure 1j; Figure S6, Supporting Information) further substantiated the presence of Gd^3 +^ ions in MOFs.^[^ ^30^ ^]^ Collectively, we successfully synthesized Gd‐BBDC MOFs with Gd^3 +^ ions as the metal nodes and BBDC as the functional ligand. Our findings indicate that the boric acid groups in the ligands did not participate in chelation with Gd^3 +^, leaving the boric acid sites available for binding to the bacterial surface's cis‐o‐dihydroxy structures,^[^ ^31^ ^]^ thus enabling specific bacterial targeting.

To further corroborate the coordination environment of Gd atom in Gd‐BBDC1.25, X‐ray absorption near‐edge structure (XANES) characterization and extended X‐ray absorption fine structure (EXAFS) analysis were conducted. Figure 1k shows Gd L_3_ edge XANES spectra of Gd‐BBDC1.25, Gd foil, and standard Gd_2_O_3_. The intensity of the Gd L_3_ edge in Gd‐BBDC1.25 is comparable to Gd_2_O_3_, indicating the existence of Gd^3 +^ in Gd‐BBDC1.25 MOFs. The Fourier transform (FT) EXAFS spectra of Gd‐BBDC1.25 exhibited a dominant Gd─O coordination at 1.9 Å (Figure 1l), and no significant signal corresponding to Gd−B/Gd−Gd coordination was detected. The Gd‐BBDC1.25 Gd L‐edge EXAFS plot well matches with the fitting data in R space and K space (Figure 1l; Figure S7, Supporting Information), and the detailed parameters were listed in Table S2 (Supporting Information). Additionally, wavelet transform (WT)‐EXAFS with high resolution in both R‐ and k‐space was performed (Figure 1m). The WT plots of Gd‐BBDC1.25 displayed a maximum of 4.6 Å^−1^, which was assigned to Gd−O coordination.^[^ ^32^ ^]^ No signal for Gd−B/Gd−Gd coordination was found, further demonstrating that Gd is coordinated to O atom of carboxylic acid in BBDC ligand.

### 2.2 Enhanced Longitudinal Relaxivity by Geometrical Confinement and Mechanism Study

**Figure 2:**
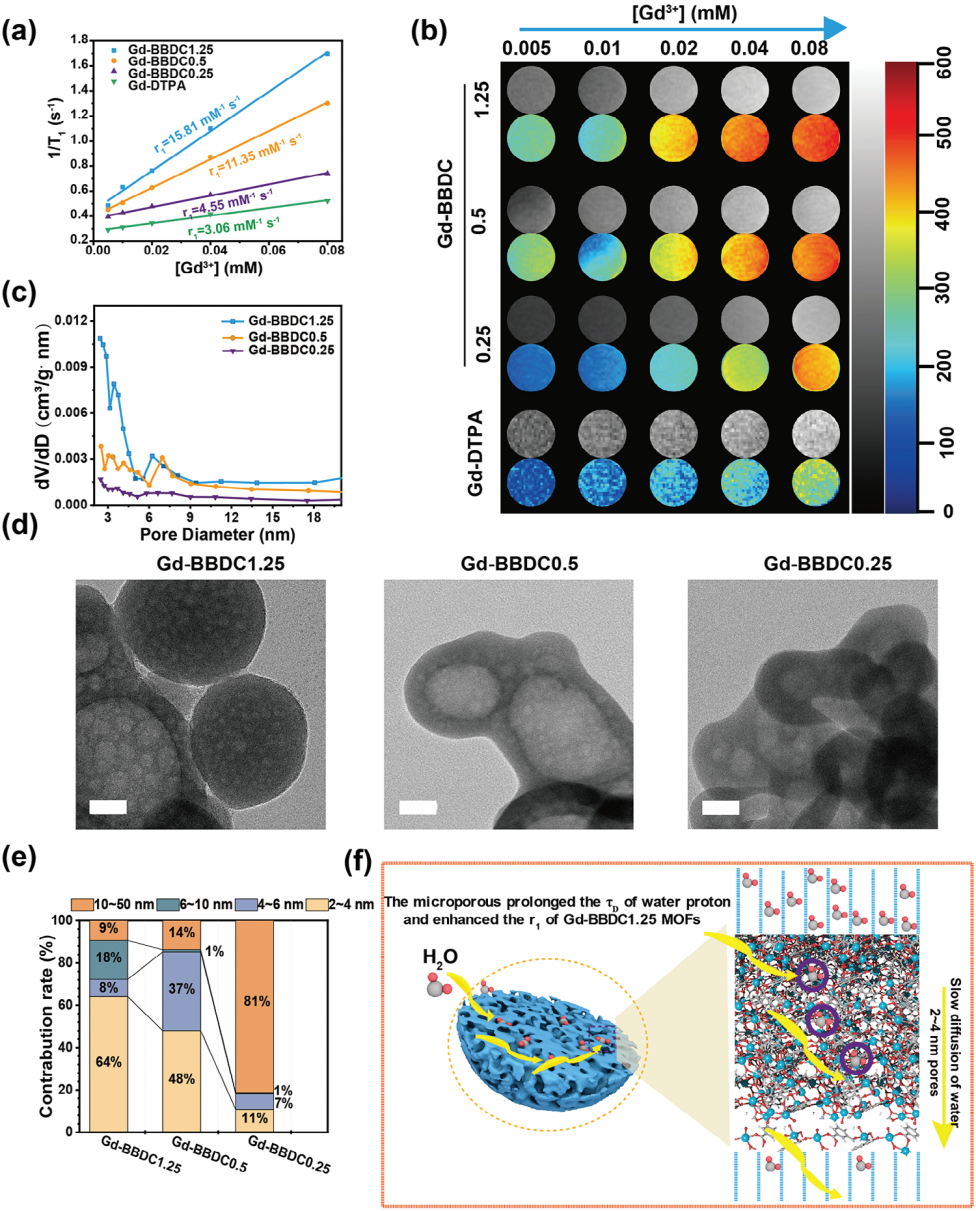
The longitudinal relaxivity measurement of the Gd‐BBDC MOFs and the mechanism study of the enhanced longitudinal relaxivity of Gd‐BBDC1.25. a) The longitudinal relaxivity of Gd‐BBDC1.25, Gd‐BBDC0.5, Gd‐BBDC0.25 and Gd‐DTPA. b) T_1_‐weighted MR images of three Gd‐BBDC MOFs and Gd‐DTPA with different Gd^3 +^ concentrations. c) Pore‐size distributions of three Gd‐BBDC MOFs. d) HRTEM images of three Gd‐BBDC MOFs. Scale bar: 20 nm. e) The pore sizes proportion of three Gd‐BBDC MOFs. f) Schematic illustration of the Gd‐BBDC1.25 with enhanced longitudinal relaxivity.

We evaluated the longitudinal relaxivity of various Gd‐BBDC MOFs using a Bruker Minispec MQ60 NMR analyzer operating at a 1.41 T magnetic field and 37 °C. The relaxivity (r_1_) were determined to be 15.81 mM^−1^ s^−1^ for Gd‐BBDC1.25, 11.35 mM^−1^ s^−1^ for Gd‐BBDC0.5, and 4.55 mM^−1^ s^−1^ for Gd‐BBDC0.25 (**Figure** **2**a). Notably, Gd‐BBDC1.25 demonstrated significantly enhanced T_1_‐weighted MRI brightness compared to Gd‐BBDC0.5 and Gd‐BBDC0.25 at equivalent gadolinium concentrations (Figure 2b). It can be seen that the r_1_ of the resulting MOF increases with the increase of BBDC concentration. However, an increase in BBDC concentration to 2.5 mmol L^−1^ resulted in a reduced r_1_ of 6.99 mM^−1^ s^−1^ for Gd‐BBDC2.5 (Figure S8, Supporting Information), potentially due to its non‐uniform size distribution. According to the outer‐sphere mechanism of the Solomon‐Bloomberg‐Morgan (SBM) relaxivity theory, which is universal to nanomaterials, identifies that the diffusion correlation time (τ_D_) of the water molecules surrounding the contrast agents (CAs) is the dominant contributor to the relaxation rates. An increase of the τ_D_ for water molecules (outer‐sphere effect), which are geometrically confined and forced to interact longer with paramagnetic center atom of the CAs. In porous structures, the diffusion coefficient of confined water molecules, Dcw, significantly smaller than the free bulk value (Dw ≈ 3.3 × 10^−9^ m^2^ s^−1^).^[^ ^33^ ^]^ Moreover, the Dcw was reported to decrease with decreased pore size.^[^ ^21^ ^]^ Based on this, we postulate that the relaxation of Gd‐BBDC MOFs is closely associated with their pore architecture. Specifically, we propose that the variation in relaxation of the Gd‐BBDC1.25, Gd‐BBDC0.5, and Gd‐BBDC0.25 may be induced by the differential pore composition. High‐resolution transmission electron microscopy (HRTEM) images revealed the hierarchical porous microstructure of Gd‐BBDC MOFs (Figure 2d). Nitrogen adsorption‐desorption isotherms displayed type IV curves with capillary condensation at P/P_0_ = 0.6–0.8, indicative of the coexistence of micropores and mesopores within the Gd‐BBDC MOFs (Figure S9, Supporting Information). This was corroborated by the pore size distribution curve (Figure 2c), which showed that the pore sizes predominantly consisted of 2–4, 4–6, 6–10, and 10–50 nm. The Gd‐BBDC1.25 exhibited the highest proportion of 2–4 nm pores. A higher proportion of 2–4 nm pores in Gd‐BBDC1.25 implies a more pronounced restriction on water molecule diffusion, leading to an enhanced longitudinal relaxivity.

**Figure 3:**
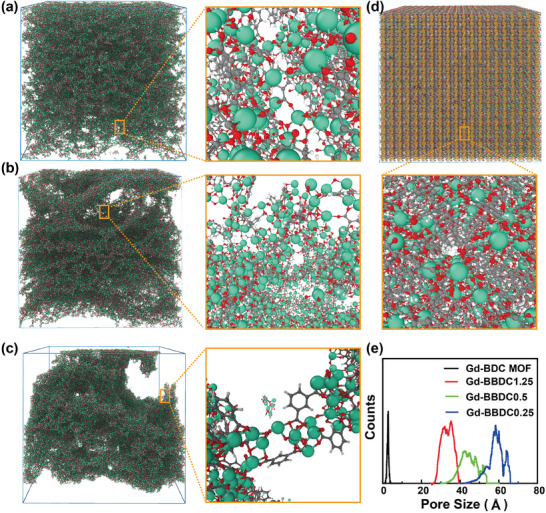
The perspective configurations of Gd‐BDC MOFs with different levels of ligand missing. The cross‐sectional slices of typical snapshots of Gd‐BDC MOFs with a) 50%, b) 75%, c) 90%, and d) 0% ligand missing and corresponding enlarged snapshots. e) The simulated pore‐size distributions of Gd‐BDC MOFs with different ligands missing.

The effect of ligand content on the porous distribution of Gd‐BBDC MOFs was confirmed by the classical molecular dynamics (MD) simulations through the phase‐pure Gd_2_(BDC)_3_(H_2_O)_2_ MOF crystals from the molecular level. Here, the Gd_2_(BDC)_3_(H_2_O)_2_ MOF crystal belongs to the monoclinic crystal system with a space group of P21/N and has lattice constants of a = 13.362 Å, b = 14.475 Å, c = 13.568 Å, and α = γ = 90°, β = 104.058°. MD models are constructed by replicating 20 × 20 × 20 unit‐cells with 0.0% (Gd_2_(BDC)_3_(H_2_O)_2_MOF), 50% (simulated Gd‐BBDC1.25), 75% (simulated Gd‐BBDC0.5) and 90% (simulated Gd‐BBDC0.25) BDC ligand are randomly removed to study the porosity property. The specific MD procedure is described in the Experiments and Methods section. The Gd_2_(BDC)_3_(H_2_O)_2_ MOF crystal with different fractions of ligand removed shows distinct structural configurations. It is observed that the Gd_2_(BDC)_3_(H_2_O)_2_ MOF maintains a perfect crystalline structure with uniform distribution of ligand and Gd atoms. As the fraction of ligand removed increases, the distribution of ligand and Gd atoms in the MD box becomes more pronounced, leading to amorphization and the formation of nanovoids (**Figure** **3**b,c). In addition, the simulated pore size distribution data demonstrated that as the ligand content decreased, the pore gaps became larger, and the simulated radial distributions of 50% (simulated Gd‐BBDC1.25), 75% (simulated Gd‐BBDC0.5) and 90% (simulated Gd‐BBDC0.25) BDC ligand randomly missing were 2–4, 2.5–5, and 4–6.3 nm, respectively (Figure 3e). The results of the simulated pore size distribution of Gd_2_(BDC)_3_(H_2_O)_2_ MOF with different ligands missing were in agreement with the experimental results of the nitrogen adsorption and desorption isotherms (Figure 2c).

### 2.3 Synthesis and Characterization of Ag@Gd‐BBDC1.25 MOFs

**Figure 4:**
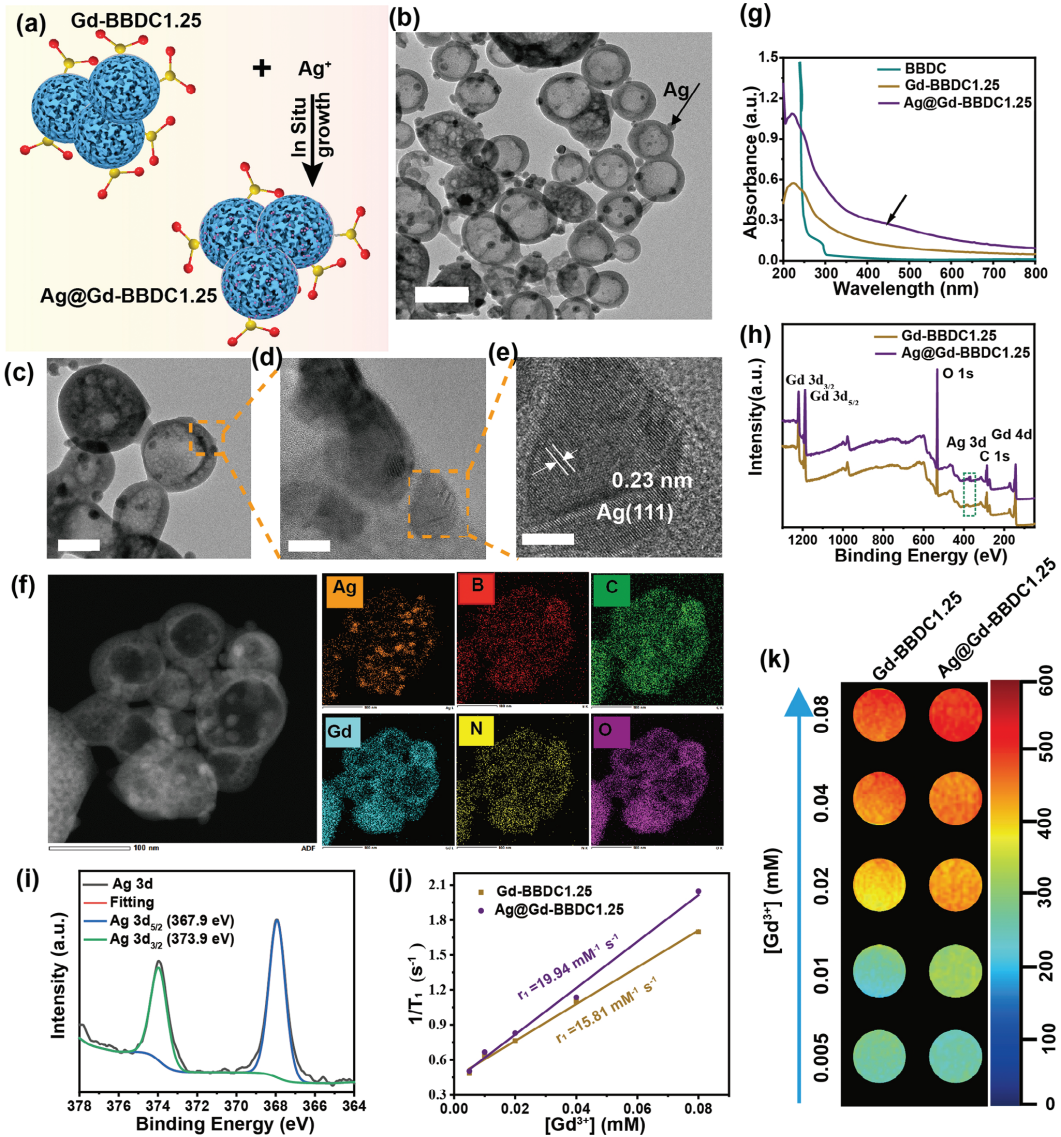
Characterization of the Ag@Gd‐BBDC1.25 nanoprobe. a) Schematic diagram of the one‐step synthesis of Ag@Gd‐BBDC1.25 using in situ reduction method. b) Typical low magnification TEM image of Ag@Gd‐BBDC1.25, scale bars: 200 nm. c,d) Enlarged TEM images of Ag@Gd‐BBDC1.25, scale bars: 50 nm; 10 nm. e) HRTEM image and Ag NPs crystal lattice on Gd‐BBDC1.25 NPs in d), scale bars: 5 nm. f) HAADF image of Ag@Gd‐BBDC1.25 and corresponding mapping of C, Ag, N, B, Gd, and O elements in Ag@Gd‐BBDC1.25, respectively. g) UV‐vis absorption spectra of BBDC, Gd‐BBDC1.25 and Ag@Gd‐BBDC1.25. h) XPS spectrum of Gd‐BBDC1.25 and Ag@Gd‐BBDC1.25. i) High‐resolution XPS spectra for Ag 3d of Ag@Gd‐BBDC1.25. j) Longitudinal Relaxivities of Gd‐BBDC1.25 and Ag@Gd‐BBDC1.25. k) T_1_‐weighted MRI images of Gd‐BBDC1.25 and Ag@Gd‐BBDC1.25 at different Gd^3 +^ concentrations.

Silver nanoparticles (Ag NPs) were successfully integrated into Gd‐BBDC1.25 MOFs via in situ reduction, preserving the hierarchical porous structure and maintaining the size of MOFs (≈90 nm), as shown in **Figures** **4**a–c and S10 (Supporting Information). Utilizing the identical synthesis procedure in the absence of MOF, Ag NPs with uneven diameters were produced (Figure S11, Supporting Information). HRTEM confirmed the (111) crystal planes of 10 nm Ag NPs with a 0.23 nm lattice spacing (Figure 4d,e) in MOFs.^[^ ^34^, ^35^ ^]^ The uniform elemental distribution in Ag@Gd‐BBDC1.25 was verified by HAADF‐STEM and elemental mapping, revealing C, O, B, Gd, and Ag (Figure 4f). Also, the Zeta‐potential of Ag@Gd‐BBDC1.25 was −10.76 mV (Figure S 12), which was more negative than that of Gd‐BBDC1.25 (−5.68 mV). Moreover, the UV‐vis absorption spectrum of Ag@Gd‐BBDC1.25 exhibited enhanced absorption (380–440 nm) due to the plasmonic effect of Ag NPs (Figure 4g).^[^ ^36^ ^]^ XPS spectra confirmed the presence of Ag^0^ NPs with peaks at 367.4 eV (Ag 3d_5/2_) and 373.4 eV (Ag 3d_3/2_) (Figure 4h,i).^[^ ^37^ ^]^ XRD patterns indicated that the crystal structure of Gd‐BBDC1.25 MOFs was unaffected by Ag NP incorporation (Figure S13, Supporting Information). The r_1_ of Ag@Gd‐BBDC1.25 (19.94 mM^−1^ s^−1^) was found to be higher than that of Gd‐BBDC1.25 (15.81 mM^−1^ s^−1^), and MR images demonstrated significantly improved brightness at equivalent Gd concentrations, possibly due to the increased τ_R_ post‐Ag NP in situ reduction (Figure 4j,k).

### 2.4 Peroxidase‐Like Activity and Enhanced ROS Generation of Ag@Gd‐BBDC1.25

**Figure 5:**
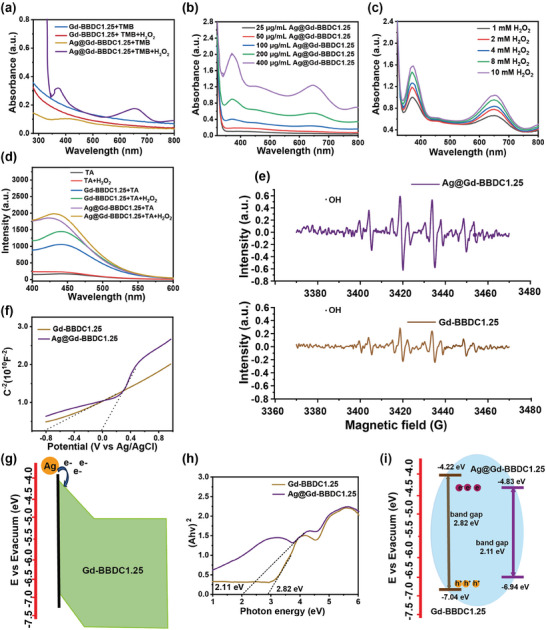
ROS generation performance and POD‐like catalytic mechanism of the Ag@Gd‐BBDC1.25 nanoprobe. a) The UV‐vis absorbance spectra of TMB in different reaction systems: Gd‐BBDC1.25 + TMB, Gd‐BBDC1.25 + H_2_O_2_ + TMB, Ag@Gd‐BBDC1.25 + TMB, and Ag@Gd‐BBDC1.25 + H_2_O_2_ + TMB. b) The UV‐vis absorbance spectra of TMB with varied concentrations of Ag@Gd‐BBDC1.25. c) The UV‐vis absorbance spectra of TMB with varied concentrations of H_2_O_2_. d) Fluorescence spectra of different reaction systems: TA, H_2_O_2_ + TA, Gd‐BBDC1.25 + TA, Gd‐BBDC1.25 + H_2_O_2_ + TA, Ag@Gd‐BBDC1.25 + TA and Ag@Gd‐BBDC1.25 + H_2_O_2_ + TA. e) Detection of POD‐like activity of Gd‐BBDC and Ag@Gd‐BBDC1.25 through TA and H_2_O_2_. f) M‐S plots of Gd‐BBDC1.25 and Ag@Gd‐BBDC1.25. e) ESR spectra of hydroxyl radicals of Gd‐BBDC1.25 and Ag@Gd‐BBDC1.25. g) Schematic diagram of the spontaneous flow of electrons over the Schottky barrier. h) Band gaps of Gd‐BBDC1.25 and Ag@Gd‐BBDC1.25 from UV‐visible diffuse reflection spectra. i) Schematic diagram of the energy band structure.

The resulting Ag@Gd‐BBDC1.25 demonstrated enhanced peroxidase (POD)‐like activity, as evidenced by the catalytic oxidation of 3,3′,5,5′‐tetramethybenzidine (TMB) in the presence of H_2_O_2_, with absorbance peaks at 625 nm (**Figure** **5**a). The Gd‐BBDC1.25 exhibits a low POD‐like activity in the presence of H_2_O_2_, while Ag@Gd‐BBDC1.25 presents significant POD‐like catalytic performance even at 1 mM H_2_O_2_ (Figure 5b,c). The decomposition of H_2_O_2_ into hydroxyl radicals (•OH) by Ag@Gd‐BBDC1.25 was confirmed by both fluorescence assays using characteristic fluorescence signal of 2‐hydroxy terephthalic acid (TA) at 435 nm and electron spin resonance (ESR) spectroscopy (Figure 5d,e), indicating superior POD‐like activity due to the presence of Ag NPs.

The catalytic mechanism of nanoenzyme can generally be summarized as an electron transfer process,^[^ ^38^ ^]^ We hypothesize that in situ reduction of Ag NPs on the surface of Gd‐BBDC1.25 may form a Schottky junctions at the interface to accelerate charge transfer. The positive spectral slope of the Mott‐Schottky (M–S) curve analysis confirmed that the n‐type Schottky heterojunctions formed at the interface of Ag NPs and Gd‐BBDC1.25 MOFs,^[^ ^39^, ^40^ ^]^ the electrons on the Ag NPs would spontaneously flow towards the Gd‐BBDC1.25 MOFs. Some of the free electrons can be captured by the surrounding oxygen to generate toxic ROS during the process (Figure 5f,g). In addition, from the M‐S spectra, the flat band potentials of Gd‐BBDC1.25 and Ag@Gd‐BBDC1.25 were calculated to be −0.18 and −0.43 eV (vs RHE), respectively. In general, the potential of conduction band (CB) is negative than 0.1 compared to the flat band of the n‐type semiconductor.^[^ ^41^ ^]^ Therefore, the CB potentials of Gd‐BBDC1.25 and Ag@Gd‐BBDC1.25 were −0.28 and −0.33 eV (vs RHE), respectively. In addition, the forbidden band gap of Gd‐BBDC1.25 and Ag@Gd‐BBDC1.25 were 2.82 and 2.11 eV, calculated from the UV‐visible diffuse reflection spectra (UV‐vis DRS) (Figure S14, Supporting Information) according to the Tauc plot method, the narrowed band gap of Ag@Gd‐BBDC1.25 indicated that the Ag NPs increased the hole concentrations and catalytic ability (Figure 5h,i).^[^ ^39^ ^]^ The Tauc method and corresponding calculation formula are listed in experimental part. Collectively, these findings suggest that Ag NPs modified on Gd‐BBDC1.25 MOFs accelerated the charge transfer at the interface, thus enhancing its POD‐like catalytic activity.

### 2.5 In Vitro Anti‐Bacterial Effect Evaluation of Ag@Gd‐BBDC1.25

**Figure 6:**
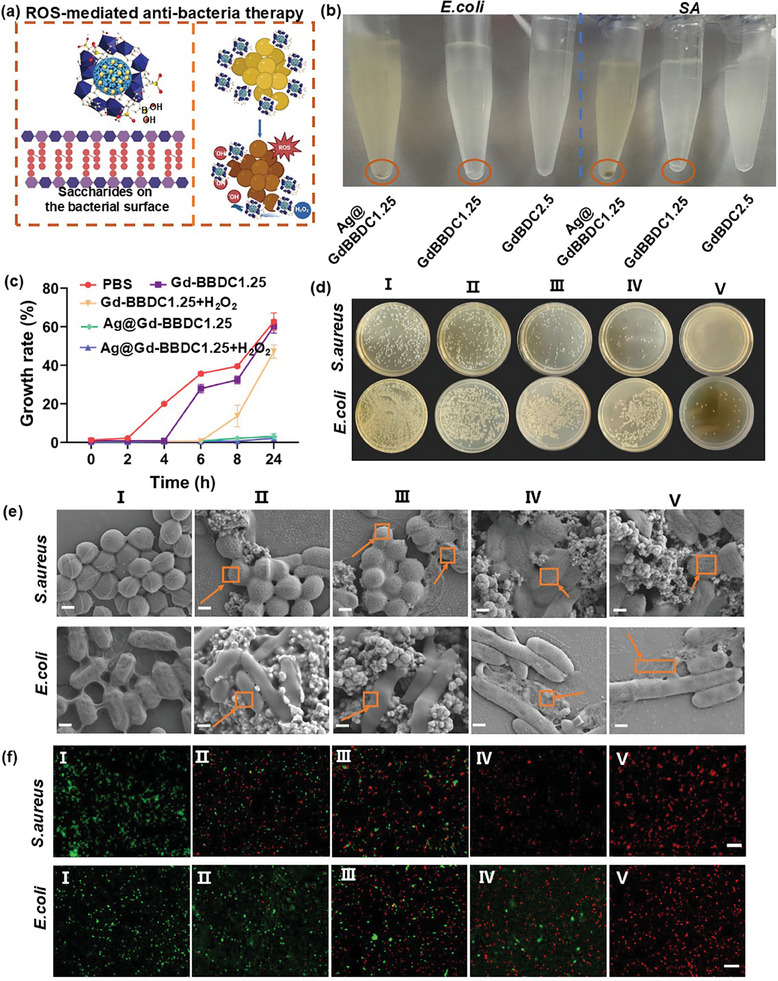
Antibacterial performance of the probes. a) Schematic illustration of the anti‐bacteria mechanism of Ag@Gd‐BBDC1.25‐mediated ROS anti‐bacteria therapy. b) Photographs of the aggregations of *E. coli* and *S. aureus* induced by Gd‐BBDC1.25 and Ag@Gd‐BBDC1.25. c) Growth rate of *S. aureus* treated with different groups during 24 h. All of the data are presented as the mean ± SD, *n* = 3. d) Plate photographs of *S. aureus* and *E. coli* forming colonies after 24 h treated with different groups. e) SEM images of *E. coli* and *S. aureus* treated by (I) PBS, (II) Gd‐BBDC1.25, (III) Gd‐BBDC1.25 + H_2_O_2_, (IV) Ag@Gd‐BBDC1.25, (V) Ag@Gd‐BBDC1.25 + H_2_O_2_, scale bar: 500 nm. f) Fluorescence images of *E. coli* and *S. aureus* that have been stained by SYTO9 and PI dyes in different treatments (I) PBS, (II) Gd‐BBDC1.25, (III) Gd‐BBDC1.25 + H_2_O_2_ (IV) Ag@Gd‐BBDC1.25 (V) Ag@Gd‐BBDC1.25 + H_2_O_2_, scale bar: 10 µm. (green fluorescence: STYO9 representing live bacteria, red fluorescence: PI representing dead bacteria).

First, the boronic acid moiety of the BBDC ligand in the Ag@Gd‐BBDC1.25 MOFs was covalently linked to the cis‐o‐dihydroxy structure present on the bacterial surface, leading to the aggregation of the Ag@Gd‐BBDC1.25 nanohybrids on the bacterial surface.^[^ ^31^ ^]^ In addition, the prepared Ag@Gd‐BBDC1.25 nanohybrids showed superior POD‐like activity and could effectively promote the generation of toxic ·OH at lower concentrations of H_2_O_2_ (100 × 10^−6^ M). And reactive oxygen species (ROS) such as H_2_O_2_, superoxide radicals, and hydroxyl radicals were shown to have significant anti‐bacterial effects.^[^ ^42^ ^]^ The schematic illustration of the antibacterial mechanism of Ag@Gd‐BBDC1.25 is shown in **Figure** **6**a. We verified the bacteria‐targeting ability of Ag@Gd‐BBDC1.25 by observing the aggregation behavior of bacteria. We found Ag@Gd‐BBDC1.25 and Gd‐BBDC1.25 induced more significant aggregation in both *S. aureus* and *E. coli* than did Gd‐BDC2.5 (Figure 6b), attributing to the bacterial‐targeting property of boric acid on Ag@Gd‐BBDC1.25 and Gd‐BBDC1.25. Next, the in vitro antibacterial effects of Ag@Gd‐BBDC1.25 combined with H_2_O_2_ were evaluated against *Staphylococcus aureus* (*S. aureus*) and *Escherichia coli* (*E. coli*) using the dilution‐plate method (Figure S15, Supporting Information). Compared to controls (Gd‐BBDC1.25, Gd‐BBDC1.25 + H_2_O_2,_ Ag@Gd‐BBDC1.25) (100 µg mL^−1^), the bacterial survival percentage of *S. aureus* and *E. coli* treated by Ag@Gd‐BBDC1.25 + H_2_O_2_ were significantly reduced to 0.53% ± 0.05% and 30% ± 0.89%, respectively (Figure S15, Supporting Information). Growth inhibition performance of Ag@Gd‐BBDC1.25 probe was further confirmed by reduced optical density (OD600nm) of bacterial suspensions during 24 h (Figure 6c; Figure S16, Supporting Information), decreased turbidity (Figure S17, Supporting Information), and diminished colony formation (Figure 6d). SEM revealed membrane disruptions in bacteria treated by Ag@Gd‐BBDC1.25 probe (Figure 6e), and live/dead staining demonstrated a substantial increase in dead cells after treatment with Ag@Gd‐BBDC1.25 and Ag@Gd‐BBDC1.25 + H_2_O_2_, indicated by red fluorescence (Figure 6f), due to the generation of toxic hydroxyl radicals. These results confirmed the significantly improved antibacterial activity of Ag@Gd‐BBDC1.25, particularly in combination with H_2_O_2_. To investigate the Ag ^+^ release behavior, the time‐dependent cumulative release profiles were studied. 5 mg of Ag@Gd‐BBDC1.25 was dispersed in 25 mL of PBS or 1 mM H_2_O_2_. The resulting mixture was kept at 37 °C. At each time point, 1mL of mixed solution was collected and centrifuged at 8000 rpm for 10 min to separate the Ag@Gd‐BBDC1.25, and the centrifuged supernatant containing dissociative Ag^+^ was obtained. ICP‐MS analysis of the released Ag^+^ in the supernatant indicated the presence of only 58 ppb dissolved Ag ^+^ in the PBS solution and 72 ppb dissolved Ag^+^ in the H_2_O_2_ solution after 24 h treatment (Figure S18, Supporting Information). In the literature, the toxicity of silver ions is a major concern, as a level of accumulated silver of 25–30 g in 6 months has been observed in human patients with argyria.^[^ ^43^, ^44^ ^]^ Therefore, the detected Ag^+^ concentration in PBS/H_2_O_2_ is much lower than the dose that could cause a harmful effect on the human body.

### 2.6 In Vivo MRI of Bacterial Infection and In situ Antibacterial Therapy

**Figure 7:**
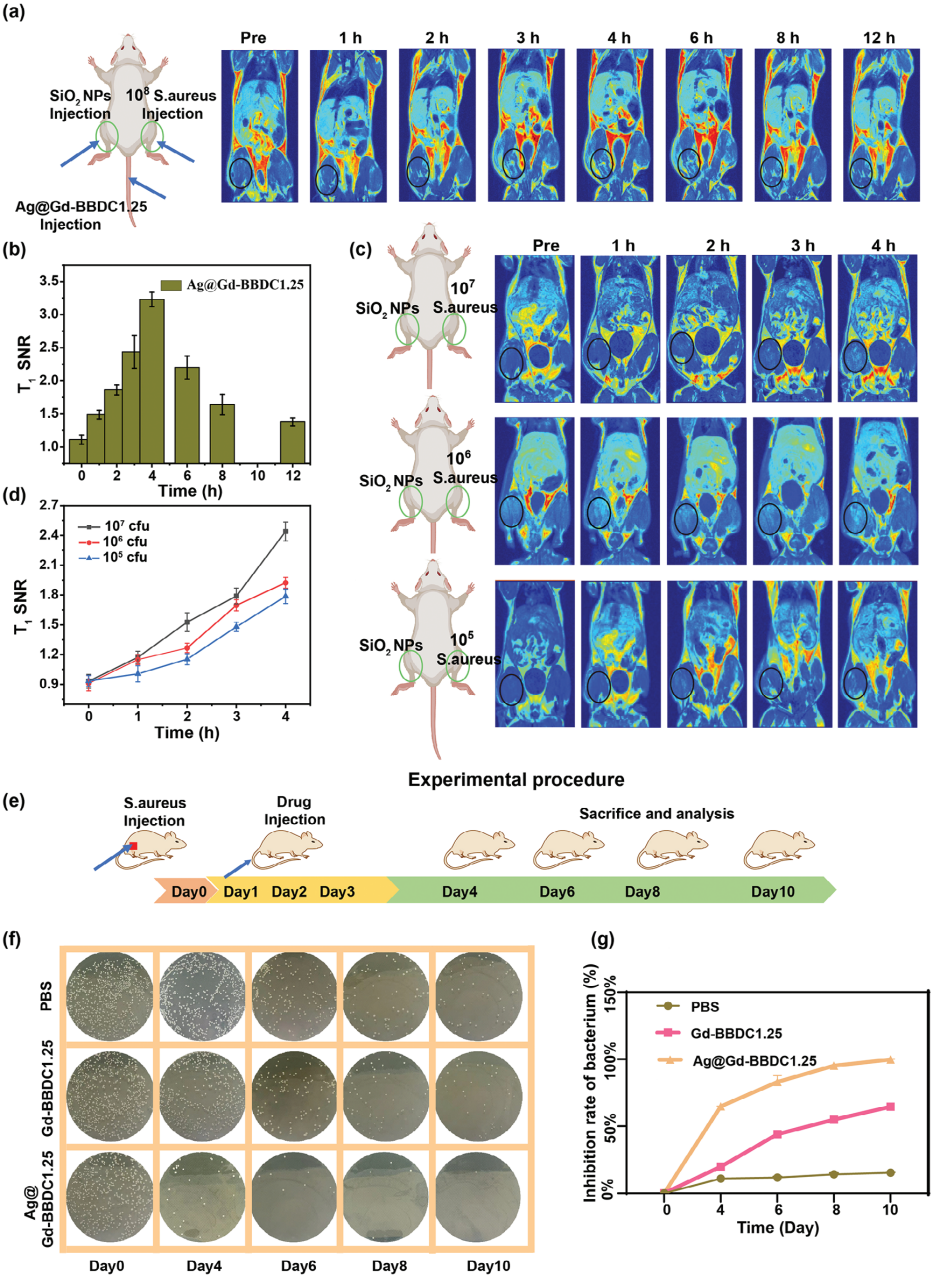
Ag@Gd‐BBDC1.25 probe for MR imaging of bacterial infection site in vivo and ROS‐mediated anti‐bacterial therapy. a) T_1_‐weighted MR images of *S. aureus*‐induced myositis mice in the right thigh muscle after injection of Ag@Gd‐BBDC1.25 probe. b) T_1_‐weighted MRI signal intensity of 10^8^ cfu *S. aureus* infected myositis mice. All data are presented as the mean ± SD, *n* = 3. c) T_1_‐weighted MR images of mice induced with various concentrations of *S. aureus* in the right thigh muscle before and after injection of the probe. d) T_1_‐weighted MRI intensity of different concentrations of *S. aureus* infected myositis mice. All data are presented as the mean ± SD, *n* = 3. e) Schematic illustration of the Ag@Gd‐BBDC1.25‐mediated treatment procedure for bacterial elimination. Bacterial colony photos f) and inhibition rate g) of *S. aureus* harvested from the infection sites of mice with different treatments at 4, 6, 8, and 10 days. All data are presented as the mean ± SD, *n* = 3.

We conducted in vivo MRI to assess the bacterial targeting of Ag@Gd‐BBDC1.25 probe. 10^8^ cfu *S. aureus* was injected into the thigh muscle of mice to establish a myositis model and the corresponding Hematoxylin and eosin (H&E) images of thigh muscle of mice has shown in Figure S19 (Supporting Information), while silica nanoparticles (SiO_2_ NPs) induced sterile inflammation in the contralateral leg. After intravenous injection of Ag@Gd‐BBDC1.25 probe, MR images of the hind limbs were acquired at various times post‐injection, with peak signal intensity observed at 4 h, indicative of targeted probe accumulation at infection sites (**Figure** **7**a,b). And meanwhile, intravenous injection of clinical gadolinium‐based contrast agent Gd‐DTPA as the control, we found that there is no MRI signal at the infected site during 2 h (Figure S20, Supporting Information). To determine the detection limit of the Ag@Gd‐BBDC1.25 probe, we created three groups of bacterial‐infected myositis models with different concentrations of *S. aureus* (10^5^, 10^6^, and 10^7^ cfu). The in vivo T_1_‐weighted MR images of the three groups are shown in Figure 7c, and the corresponding MR image signal intensities at the infection site are shown in Figure 7d. We found that the MR image signal intensity was positively correlated with *S. aureus* concentration and the MR image signals were detectable with bacteria concentrations as low as 10^5^ cfu.

The antibacterial efficacy of Ag@Gd‐BBDC1.25 probe was evaluated in vivo using *S. aureus*‐infected myositis models, divided into PBS, Gd‐BBDC1.25, and Ag@Gd‐BBDC1.25 treatment groups. Mice in each group were sacrificed on different days (4, 6, 8, and 10 days) and *S. aureus* infection sites were taken and homogenized for analysis (Figure 7e). The number of bacteria at infection sites in vivo was counted by standard plate count method (Figure 7f). The inhibition rate of different probes indicated that the Ag@Gd‐BBDC1.25 probe has an excellent antibacterial effect (Figure 7g). Biocompatibility of Ag@Gd‐BBDC1.25 was assessed through blood, liver, and kidney biomarkers and histopathology. It showed no significant toxicity, with biochemical and blood routine indexes remaining within normal ranges post intravenous injection at 30 mg kg^−1^ (Figure S21, Supporting Information). Histological analysis of major organs revealed no significant infection or inflammation, suggesting low toxicity of the probe (Figure S22, Supporting Information).

## 3 Conclusion

We successfully integrate the bacteria‐targeting capability of the BBDC ligand with Gd^3 +^ to construct hierarchically porous Gd‐BBDC MOFs. The subsequent interfacial engineering with Ag NPs results in the formation of a theranostic Ag@Gd‐BBDC1.25 nanoprobe. A unique feature of the Gd‐BBDC MOFs is the boric acid group of the BBDC ligand, which serves as an ideal targeting element for bacterial binding, covalently linked to the cis‐o‐dihydroxy structures present on the bacterial surface. The porous structure of the Gd‐BBDC MOFs is tunable, with the largest 2–4 nm pores fraction in Gd‐BBDC1.25, which enhances the T_1_ relaxivity (r_1_ of 15.81 mM^−1^ s^−1^) due to the increased the water diffusion correlation time τ_D_. The Gd‐BBDC MOFs exhibit antibacterial activity by generating ROS through POD‐like catalytic reactions. To improve the catalytic performance, Ag NPs were in situ reduced onto the Gd‐BBDC MOFs, creating interfacial Schottky heterojunctions for amplifying free electron generation that can be captured by the surrounding oxygen, leading to increased ROS production. Ag@Gd‐BBDC1.25 MOFs demonstrate both bacteria‐targeted MR imaging and bactericidal functions in vivo. The enhanced MRI signal intensity is attributed to the excellent T_1_ relaxivity of the nanoprobe and its targeted accumulation at the site of bacterial infection. The nanoprobe is capable of detecting bacterial infections in vivo at concentrations as low as 10^5^ cfu of *S. aureus*. Moreover, the accumulated Ag@Gd‐BBDC1.25 nanoprobe realizes ROS‐mediated antibacterial therapy in situ through its POD‐like catalytic activity.

## 4 Experimental Section

### Materials

1‐Borono‐3,5‐benzenedicarboxylic acid (BBDC) was provided by Shanghai Macklin Biochemical Co., Ltd. Gadolinium chloride hexahydrate (GdCl_3_⋅6H_2_O), and N, N‐dimethylformamide (DMF) were obtained by Sigma‐Aldrich Co. Ltd. Silver nitrate (AgNO_3_), Sodium borohydride (NaBH_4_), and anhydrous ethanol (CH_3_CH_2_OH) were supplied by Sinopharm Chemical Reagent Company, Ltd (Shanghai, China). The ultrapure water is prepared through a Milli‐Q Integral water purification system.

### Synthesis of Gd‐BBDC MOFs and Ag@Gd‐BBDC1.25

The boric acid‐functional Gd‐BBDC MOFs were prepared by simple solvothermal method.^[^ ^26^ ^]^ A mixture of GdCl_3_·6H_2_O (37.1 mg, 0.1 mmol) and BBDC (Four different reactant concentrations based on at 0.25, 0.5, 1.25, and 2.5 mmol L^−1^ BBDC) were vigorously stirred for 2 h in DMF/H_2_O (7:3, 8 mL) solution. Then, the mixed solution was transferred into a Teflon vessel in a stainless‐steel Teflon‐lined autoclave and heated at 120 °C for 12 h. The obtained product was cooled to room temperature, and then washed and centrifuged thoroughly with DMF and ethanol, respectively, and the Gd‐BBDC0.25, Gd‐BBDC0.5, and Gd‐BBDC1.25 products were dried at room temperature. The Ag@Gd‐BBDC1.25 heterostructure was prepared by reducing AgNO_3_ in situ on the surface of Gd‐BBDC1.25. 100 mg of Gd‐BBDC1.25 nanoparticles was dispersed in 1.5 mL of CH_3_OH. Then, 0.35 mL of AgNO_3_ (5.71 mg mL^−1^) of CH_3_OH aqueous was added to the above solution and the mixture was stirred for 2 h at room temperature, then, adding 0.5 mL of NaBH_4_ (2.5 mg mL^−1^) CH_3_OH aqueous to the above solution. The grey solid product was collected via centrifugation (10 000 rpm, 10 min) and washed three times with ethanol, and dried at 60 °C to obtain the resultant Ag@Gd‐BBDC1.25 heterostructure.

### Characterization

XRD patterns were detected using a Rigaku D/max 2500 X‐ray diffractometer (40 kV, 250 mA). TEM (FEI Tecnai F20) was used to characterize the morphology of Gd‐BBDC MOFs and Ag@Gd‐BBDC1.25, and energy dispersive X‐ray spectroscopy (EDS) was performed simultaneously to observe the element distribution. Elements of samples were further analyzed by XPS equipment (Thermo Fisher Scientific K‐Alpha). The FTIR spectra were obtained by Fourier transform infrared spectrometer (Thermo Scientific Nicolet iS20). To characterize the mesoporous structure of prepared materials, the N_2_ adsorption‐desorption isotherm and corresponding pore‐size distribution were tested (Micromeritics ASAP 2460). The UV‐vis absorption spectrum was tested with the spectrometer (Lambda750, PerkinElmer). The UV‐visible diffuse reflection spectra data was tested by UV‐vis DRS spectrometer (PE lambda 750, UV3600, Shimadzu). The Tauc plot is a method originally developed to derive the optical gap of amorphous semiconductors such as amorphous germanium or silicon. The calculation formula is as follows: (αhv)^1/m^ = B (hν‐Eg), (m = 2 for indirect bandgap, B is a constant, hv is the photon energy, h is the Planck's constant, ν for the incident photon frequency, Eg represents the semiconductor bandgap). By measuring the absorption coefficient *α*(*hν*) and plotting (αhv)^1/2^ versus photon energy *hν*, a value for the optical gap (Tauc gap) is determined.^[^ ^45^ ^]^ Gd^3 +^ concentrations were tested on ICP‐OES (Agilent 720ES, USA). The X‐ray absorption data at the Gd L‐edge of the samples were recorded at room temperature in transmission mode using ion chambers or in the fluorescent mode with silicon drift fluorescence detector at beamline BL14W1 of the Shanghai Synchrotron Radiation Facility (SSRF), China. The formation of •OH signals was further detected by ESR. To a 0.2 mL portion of the material aqueous solution (2 mg mL^−1^), 20 µL of H_2_O_2_ was added and the mixture was incubated at 37 °C for 4 h. Then 1 µL of 5,5′‐Dimethyl‐1‐pyrroine N‐oxide (DMPO) radical scavenger was added to the mixture and then the •OH signal was tested immediately.

### XAFS Data Analysis

The station was operated with a Si(111) double crystal monochromator. Data reduction, data analysis, and EXAFS fitting were performed and analyzed with the Athena and Artemis programs of the Demeter data analysis packages that utilize the FEFF6 program to fit the EXAFS data. The energy calibration of the sample was conducted through standard Gd_2_O_3_ and Gd foil, which as references were simultaneously measured. A linear function was subtracted from the pre‐edge region, then the edge jump was normalized using Athena software. The χ(k) data were isolated by subtracting a smooth, third‐order polynomial approximating the absorption background of an isolated atom. The k2‐weighted χ(k) data were Fourier transformed after applying a HanFeng window function (Δk = 1.0) for EXAFS modeling. The global amplitude EXAFS (CN, R, σ2 and ΔE0) was obtained by nonlinear fitting, with least‐squares refinement, of the EXAFS equation to the Fourier‐transformed data in R‐space, using Artemis software. EXAFS of the Gd foil are fitted and the obtained amplitude reduction factor S02 value (0.755 was set in the EXAFS analysis to determine the coordination numbers (CNs) in sample.

### MRI Measurement

Longitudinal (T_1_) relaxation times of different Gd‐BBDC MOFs contrast agents were acquired using the MQ60 NMR analyzer at a magnetic field strength of 1.4 T and 37 °C. The Gd‐BBDC MOFs and Ag@Gd‐BBDC1.25 were diluted to different concentrations (0.05, 0.1, 0.2, 0.4, 0.6, and 0.8 mM of Gd) with water and respectively placed in a 2 mL Eppendorf tube for relaxation time. The resulting T_1_ values at different concentrations were recorded and plotted as 1/T_1_ versus molar concentration of Gd, respectively. The slope of the fitted line provided the relaxivity (r_1_) of the contrast agents. Among them, the concentration of Gd ions was determined by ICP‐OES. In a digestion flask, a certain amount of material was added to 5 mL of nitric acid, and then the solution was evaporated. The residue was re‐dissolved in deionized water in a 10 mL volumetric flask for ICP measurement. The MRI performance of Gd‐BBDC MOFs and Ag@Gd‐BBDC1.25 with different concentrations was obtained on the Niumag MR imaging system (NM42‐060H‐I).

### MD Simulations

The Periodic boundary conditions (PBCs) were imposed in the three orthogonal (*x*, *y*, and *z*) directions to avoid the surface effect. To describe the atomic interaction in the Gd‐BDC system, the Universal Force Field (UFF)^[^ ^46^ ^]^ was employed, in which the atomic charges of as‐investigated systems were assigned via the repeating electrostatic potential extracted atomic charge method (REPEAT) method.^[^ ^47^ ^]^ For the non‐bonded atomic interactions in the system, the 12–6 Lennard‐Jones potential with cutoff distance of 10.0 Å is applied to describe the van der Waals (vdW) forces between atoms, while the standard Coulomb potential was utilized to mimic the electrostatic interactions that were evaluated by the particle‐particle particle‐mesh (PPPM) algorithm. Prior to MD simulations, as‐investigated systems are first structurally optimized to a local minimized configuration with energy and force tolerances of 0.00001 Kcal mol^−1^ and 0.00001 Kcal (mol·Å)^−1^, respectively. Secondly, MD simulations with 1000 000 timesteps are carried out to further relax the systems under canonical (NVT) ensemble at temperature of 800 K, in which the temperature was controlled by the Nose‐hoover thermostat. Finally, production MD simulations with 1000 000 timesteps at temperature of 300 K were performed to achieve stable structures. The dynamics of atoms in the systems follow the classical Newton's motion, in which the velocity‐Verlet algorithm was utilized to integrate the classic Newton's equation. All the MD simulations were implemented using the Large‐scale Atomic/Molecular Massively Parallel Simulator (LAMMPS) package. The geometry‐based analysis Zeo++ software^[^ ^48^ ^]^ was adopted to calculate the pore property in the Gd‐BBDC structural systems.

### Mott‐Schottky Electrochemical Measurement

The Mott−Schottky electrochemical testing of the samples was undertaken using an electrochemical workstation (CHI660E, China) with a conventional three‐electrode system, where an Ag/AgCl electrode served as the reference electrode, a platinum foil was the counter electrode, and each sample was used as the working electrode. To fabricate the working electrode, 200 µL of the mixture, each containing 4 mg of the sample, 1 mL of deionized water, and 80 µL of Nafion solution, were dropped on fluoride‐tin oxide conductor glass to form a uniform film. All measurements were processed in 0.5 m Na_2_SO_4_ solution at 10 Hz. The resulting potential applied to Ag/AgCl was converted to the RHE potential by the formula

(1) $$E_{\mathrm{RHE}}=E_{\mathrm{Ag}/\mathrm{AgCl}}+0.0591\;\mathrm{pH}+{E^{\theta }}_{\mathrm{Ag}/\mathrm{AgCl}}\;{E^{\theta }}_{\mathrm{Ag}/\mathrm{AgCl}}=0.199$$

From the Mott−Schottky diagram, the flat band potential (*E*_fb_) could be obtained, which was obtained from the x intercept of the linear region.^[^ ^40^ ^]^ The formula for calculating the minimum conduction band potential (*E*_CBM_) is *E*_CBM_ = *E*_fb_ − 0.1, where 0.1 is an empirical value. And the RHE potential can be converted to the energy level position under vacuum by the equation

(2) $$E_{\mathrm{vs}\mathrm{vacuum}}=-4.5-E_{\mathrm{RHE}}$$

### Bacterial Cultures and Preparation

The *S. aureus* and *E. coli* as representative Gram‐positive and Gram‐negative bacteria, respectively. *S. aureus* and *E. coli* were cultured in 5 mL Luria Bertani (LB) broth medium with shaking at 37 °C. After shaking for 12 h, bacteria were collected by centrifuging at 5000 rpm for 5 min and washed with saline three times. Finally, the bacteria were re‐suspended in saline and kept at 4 °C for further use.

### Bacterial Cultures and Preparation—SEM Imaging

*S. aureus* and *E. coli* as representative Gram‐positive and Gram‐negative bacteria, respectively, were selected to investigate the interaction between the NPs and bacterial cells. *S. aureus* and *E. coli* were cultured for 12 h in LB broth medium (37 °C, 120 rpm). The bacteria were diluted to a density of 10^8^ CFU mL^−1^, and 100 µL of bacterial solution was incubated with Gd‐BBDC1.25 and Ag@Gd‐BBDC1.25 NPs （200 µg mL^−1^) at 37 °C for 4 h. Afterward, the bacteria were centrifuged and washed by PBS. The collected bacteria were then immobilized with 2.5% glutaraldehyde and dehydrated by adding ethanol according to a graded series (30–100%). Finally, samples were dispersed in ethanol and dripped onto a coverslip, freeze‐dried, and observed by a scanning electron microscope (SEM, ZEISS Gemini SEM 300).

### Bacterial Cultures and Preparation—Antibacterial Activity In Vitro

*S. aureus* and *E. coli* were selected to investigate the bactericidal activity of Gd‐BBDC1.25 and Ag@Gd‐BBDC1.25 in vitro. 1 mL *S. aureus* (≈1 × 10^6^ CFU mL^−1^, dispersed in LB culture) was incubated with different concentrations of the material nanosystem (0–200 µg mL^−1^) at 37 °C for 4 h. The number of remaining bacteria was determined by plate counting to assess antibacterial ability.

### Bacterial Cultures and Preparation—Bacterial Live/Dead Assays

*S. aureus* (≈1 × 10^8^ CFU mL^−1^) was incubated as described in the previous section. After incubation, the *S. aureus* cells were collected by centrifugation at 5000 rpm for 5 min and co‐stained with propidium iodide (PI, labeled damaged or dead *S. aureus*) and 4′,6‐diamidino‐2‐phenylindole staining (SYTO9, labeled live *S. aureus*) for 30 min under dark conditions. After that, the *S. aureus* solutions were washed with PBS three times to remove excess dye. For each treatment, the dead *S. aureus* cells were observed and imaged by confocal microscopy (Nikon, Ti‐E + A1 SI).

### MRI In Vivo

Male Balb/c mice (6–8 weeks) were purchased from GemPharmatech Co., Ltd, Nanjing China. All animal experiments were carried out under the permission of Tongji University Laboratory Animal Center (SYXK (Shanghai) 2020–0002, TJAA08124102), and were performed ethically and humanely. *S. aureus*‐infected inflammation model was fabricated in the thigh muscle of each test mouse. In brief, male Balb/c mice (≈20 g) were anesthetized with isoflurane, and each of their right thigh muscles was injected with the suspension of *S. aureus*. After injection for 24 h, inflammation was observed in the muscle location. The *S. aureus*‐infected mouse model was built to evaluate in vivo MRI. To produce subcutaneous inflammation, 50 µL of *S. aureus* (≈10^8^, 10^7^, 10^6^, and 10^5^ CFU mL^−1^) in saline was subcutaneously injected into mice, and the symmetric position site was subcutaneously injected with SiO_2_ as control. After 24 h, the *S. aureus* infected mice were intravenously injected with 1 mg kg^−1^ dose of Ag@Gd‐BBDC1.25 or Gd‐BBDC1.25. At each time point, treated mice were MR imaged by a Niumag MR imaging system (NM42‐060H‐I). The images were obtained using a T_1_ sequence with the following parameters: TR/TE = 400/18.14 ms, thickness = 2.5 mm, FOV = 60 × 60 mm, flip angle = 90°. A signal‐to‐noise ratio (SNR) was obtained by analyzing regions of interest (ROIs) in the images. Contrast enhancement was assessed by an increase of SNR after injection, ΔSNR = (SNR_post_ − SNR_pre_)/SNR_pre_.

### Antibacterial Effect In Vivo

To assess the antibacterial efficacy of Ag@Gd‐BBDC1.25, *S. aureus*‐infected mice were randomly divided into three groups (*n* = 5). A suspension of *S. aureus* (10^8^ cfu) was injected into the right thigh muscle of mice, and infected mice were obtained after 24 h. Then those mice were injected with Ag@Gd‐BBDC1.25 via the tail vein (every 24 h for 3 d), and control mice received saline and Gd‐BBDC1.25 instead of Ag@Gd‐BBDC1.25. The infected muscles of mice were extracted, weighed, and homogenized and the number of bacteria was determined by plate counting method.

### In Vivo Toxicity Evaluation

The healthy male Balb/c mice were randomly divided into two groups and intravenously injected with the same volume of Ag@Gd‐BBDC1.25 or PBS (control group), respectively. After 24 h, mice were sacrificed to gather their major visceral organs (heart, liver, spleen, lung, and kidney) which were preserved in a 10% formalin solution for further histopathology analysis using a typical hematoxylin and eosin (H&E) staining assay. The H&E‐stained tissue sections were monitored including the heart, liver, spleen, lung, and kidney using an optical microscope. Albumin (ALB), alkaline phosphatase (ALP), alanine transaminase (ALT), creatinine (CREA), aspartate transaminase (AST), total protein (TP), blood urea nitrogen (BUN), and urea (UA), together with white blood cells (WBC), red blood cells (RBC), red blood cells distribution width‐standard deviation (RDW‐CV), hemoglobin (HGB), mean corpuscular hemoglobin concentration (MCHC), mean corpuscular hemoglobin (MCH), hematocrit (HCT) and lymphocyte (LYM) of all groups by using authoritative standard biochemistry test.

### Statistical Analysis

All experimental data were exhibited as mean ± standard deviation (*n* = 3), and the statistical analysis was implemented using the GraphPad Prism 10 software.

## Conflict of Interest

The authors declare no conflict of interest.

## Supporting information



Supporting Information

## Data Availability

The data that support the findings of this study are available from the corresponding author upon reasonable request.
