# Supplementary material for: Tunable Hierarchically Porous Gadolinium‐Based Metal‐Organic Frameworks for Bacteria‐Targeting Magnetic Resonance Imaging and In Situ Anti‐Bacterial Therapy
Source: Adv Sci (Weinh). 2025 Feb 20;12(15):2415209. doi: 10.1002/advs.202415209 (PMC12005816; doi:10.1002/advs.202415209)
Supplement: Supplementary file 1 — Supporting Information [file ADVS-12-2415209-s001.docx]

**Tunable Hierarchically Porous Gadolinium-based Metal-Organic Frameworks for** **Bacteria-Targeting Magnetic Resonance Imaging and** ***in-situ* Anti-bacterial Therapy**

*Youyi Yu*^1^, *Tianming Cui*^2^, *Chang Liu*^1^, *Weitao Yang*^1^, *Bingbo Zhang*^1*^

^1^Department of Radiology, Tongji Hospital, Shanghai Frontiers Science Center of Nanocatalytic Medicine, the Institute for Biomedical Engineering & Nano Science, School of Medicine, Tongji University, Shanghai 200065, China

^2^Shanghai Research Institute for Intelligent Autonomous Systems, Tongji University, Shanghai 200065, China

*Corresponding Author. Email: [bingbozhang@tongji.edu.cn](mailto:bingbozhang@tongji.edu.cn)

Keywords: bacteria targeting, theranostics, nanoprobes, magnetic resonance imaging, hierarchical porous structure

**Table S1** Overview of Gd-based MOFs and their relaxivity properties

| **MRI active component** | **Ligand** | **Particle size** | **MRI studies** | | | | **Ref** |
| --- | --- | --- | --- | --- | --- | --- | --- |
|  |  |  | **r_1_** | **r_2_** | **Mag. field (T)** | **Medium** |  |
| Gd(Ⅲ) | 1,4-benzene-dicarboxylate | Rod: ca. 400 nm× 70 nm | 35.8 | 55.6 | 3.0 | 0.1% xanthan gum | ^1^ |
| Gd(Ⅲ) | 1,2,4-benzene-tri-carboxylate | Rod: ca. 100 nm× 35 nm | 13.0 | 29.4 | 3.0 | 0.1% xanthan gum | ^1^ |
| Gd(Ⅲ) | N-(4-carboxy-benzyl)-(3,5-di-carboxyl) pyridinium bromide | 70± 8.2 nm | 13.46 | - | 3.0 | water | ^2^ |
| Gd(Ⅲ) | 5-boronobenzene-1,3-dicarboxylic acid | 168 nm | 6.24 |  | 1.2 | water | ^3^ |
| Gd(Ⅲ) | 1,4-benzene-dicarboxylate | Rod: ca. 155 nm× 30 nm | 4.9 |  | 4.7 | water | ^4^ |
| Gd(Ⅲ) | Isophthalic acid | 100±20 nm | 38 | 222 | 7.0 | 1% agarose gel | ^5^ |
| Gd(Ⅲ) | Tetrakis(4-carboxyphenyl) porphyrin | Square plates: 221± 63 nm  Thickness: 21 ± 9.4 nm | 10.04 | - | 0.5 | water | ^6^ |
| Gd(Ⅲ) | 2,2’-bipyridine-6,6’-dicarboxylate | 100 nm | 8.27 | - | 7.0 | water | ^7^ |
| Gd(Ⅲ) | 5-boronobenzene-1,3-dicarboxylic acid | 90 nm | 15.81 | - | 1.41 | Water | The article |


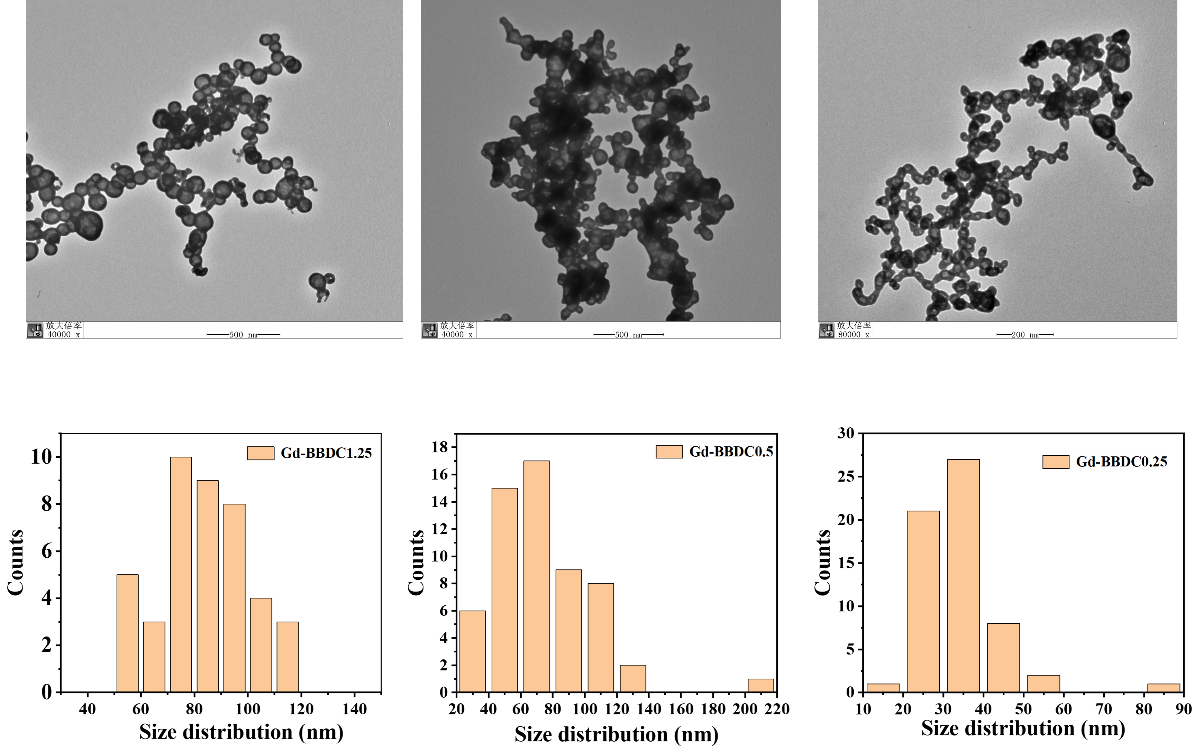


**Figure S1** TEM images of Gd-BBDC1.25, Gd-BBDC0.5 and Gd-BBDC0.25 MOFs and corresponding size distributions as analyzed by ImageJ software.





**Figure S2** TEM images of Gd-BBDC2.5 MOFs.

**
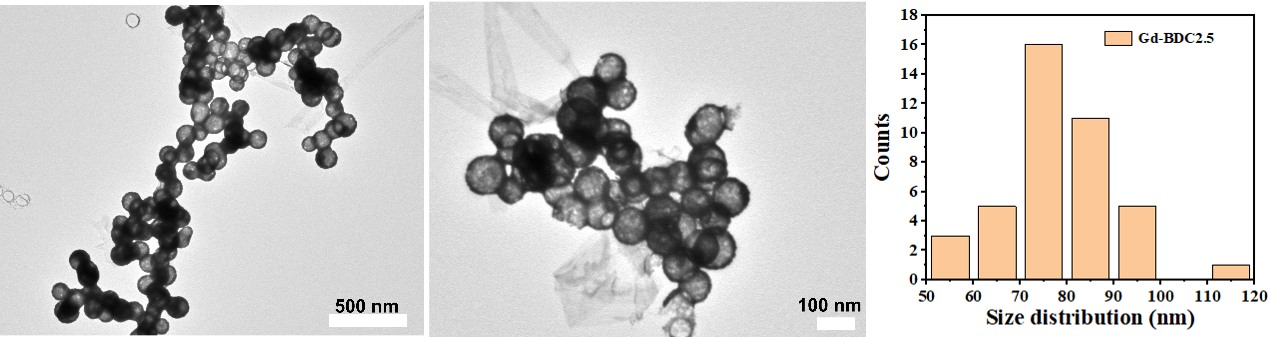
**

**Figure S3** TEM images of Gd-BDC2.5 and corresponding size distributions as analyzed by ImageJ software.


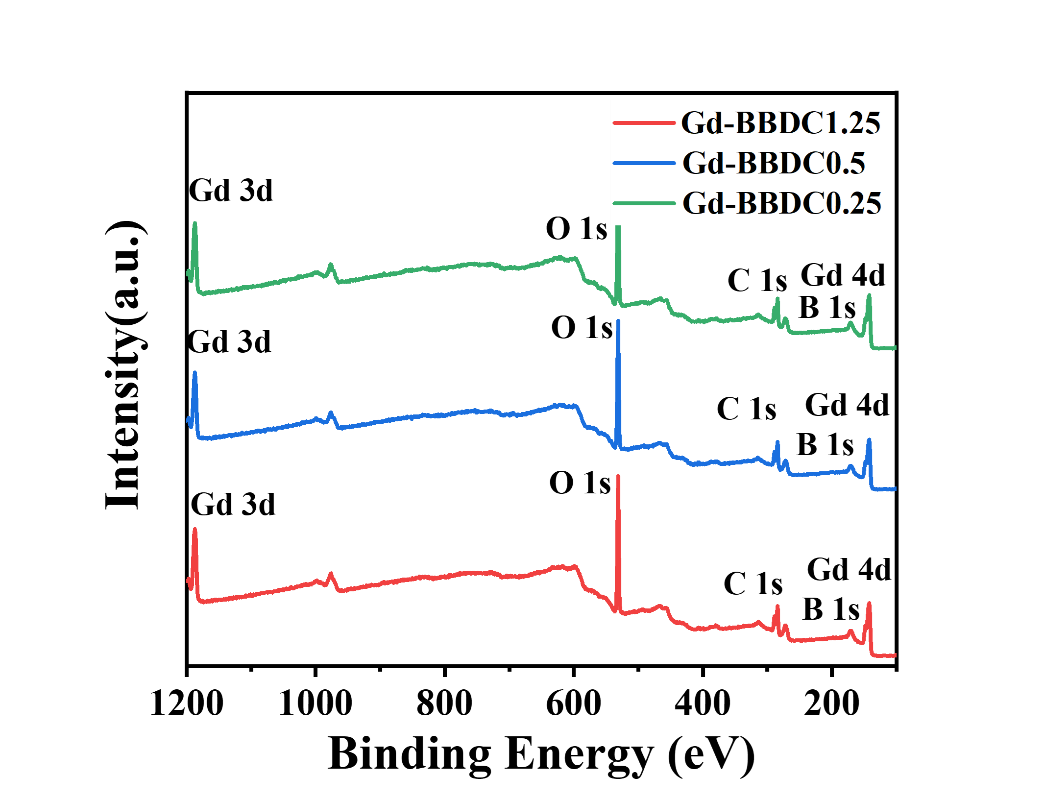


**Figure S4** XPS full spectra of Gd-BBDC1.25, Gd-BBDC0.5 and Gd-BBDC0.25 MOFs.





**Figure S5** High-resolution O1s XPS spectrum of Gd-BBDC1.25 MOFs.





**Figure S6** High-resolution Gd3d XPS spectrum of Gd-BBDC1.25 MOFs.

**Figure S7** Fourier-transforms of Gd L_3_-edge EXAFS spectra and corresponding fitting results in K space.

**Table S2** EXAFS fitting parameters at the Gd L3–edge for various samples

| **Sample** | **Shell** | ***CN^a^*** | ***R*(Å)*^b^*** | ***σ*^2^(****Å^2^)*^c^*** | **Δ*E*_0_(eV)*^d^*** | ***R* factor** |
| --- | --- | --- | --- | --- | --- | --- |
| Gd foil | Gd-Gd | 12* | 3.86±0.01 | 0.0075 | -9.6 | 0.0053 |
| Gd_2_O_3_ | Gd-O | 6* | 2.32±0.01 | 0.0072 | 2.3 | 0.0160 |
|  | Gd-Gd | 6* | 3.60±0.01 | 0.0057 | 0.9 |  |
|  | Gd-Gd | 6* | 4.11±0.01 | 0.0067 | 6.1 |  |
| Gd-BBDC1.25 | Gd-O | 7.1±0.6 | 2.43±0.01 | 0.0046 | 5.2 | 0.0138 |

*^a^CN*, coordination number; *^b^R*, distance between absorber and backscatter atoms; *^c^σ*^2^, Debye-Waller factor to account for both thermal and structural disorders; *^d^ΔE*_0_, inner potential correction; *R* factor indicates the goodness of the fit. S_0_^2^ was fixed to 0.755, according to the experimental EXAFS fit of Gd foil by fixing CN as the known crystallographic value. A reasonable range of EXAFS fitting parameters: 0.600 < *Ѕ*_0_^2^ < 1.000; *CN >* 0; *σ*^2^ > 0 Å^2^; |Δ*E*_0_| < 15 eV; *R* factor < 0.02.

**
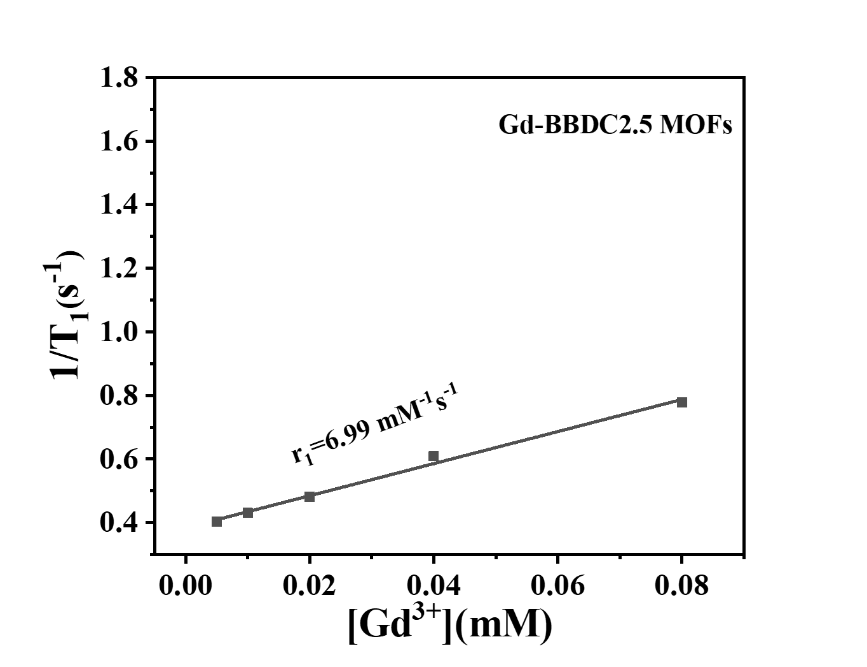
**

**Figure S8** The r_1_ of Gd-BBDC2.5





**Figure S9** Nitrogen sorption-desorption isotherms of Gd-BBDC1.25, Gd-BBDC0.5, and Gd-BBDC0.25 MOFs.

**
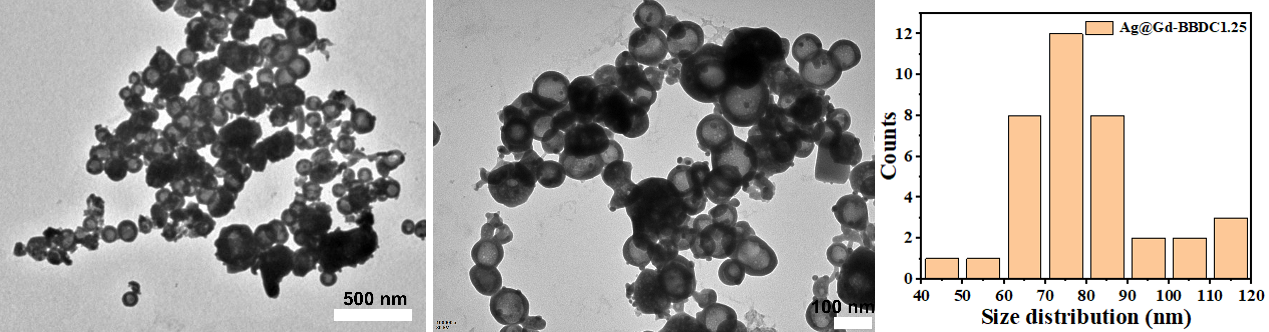
**

**Figure S10** TEM images of Ag@Gd-BBDC1.25 NPs and corresponding size distributions as analyzed by ImageJ software.

**
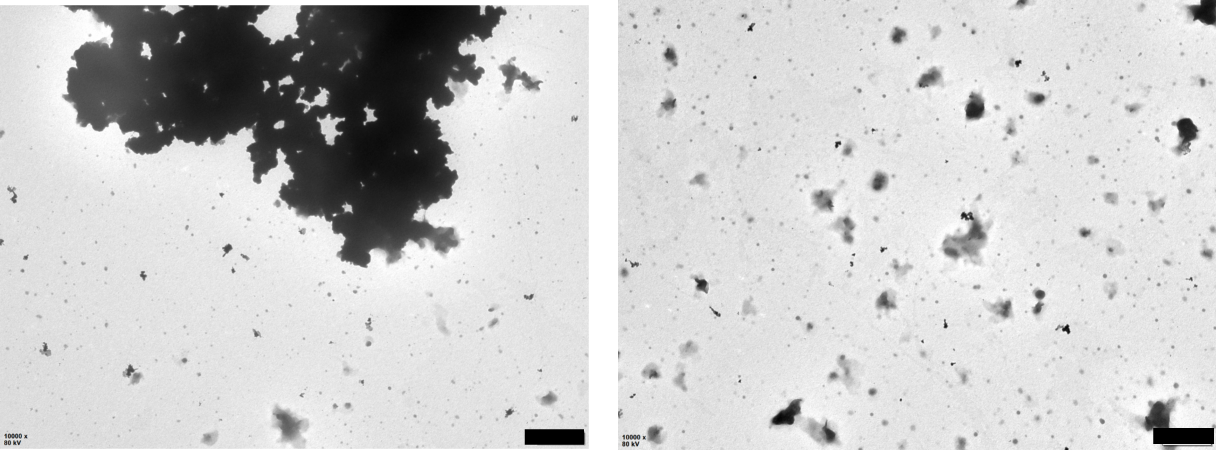
**

**Figure S11** TEM images of Ag NPs synthesized by AgNO_3_ and NaBH_4_. Scale bar:1 μm.


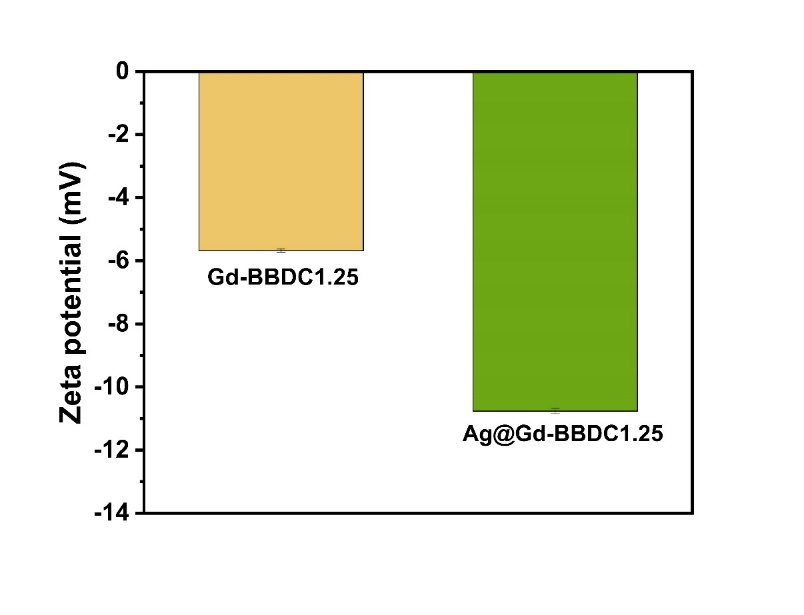


**Figure S12** The zeta potentials of Gd-BBDC1.25 and Ag@Gd-BBDC1.25 in ultrapure water.


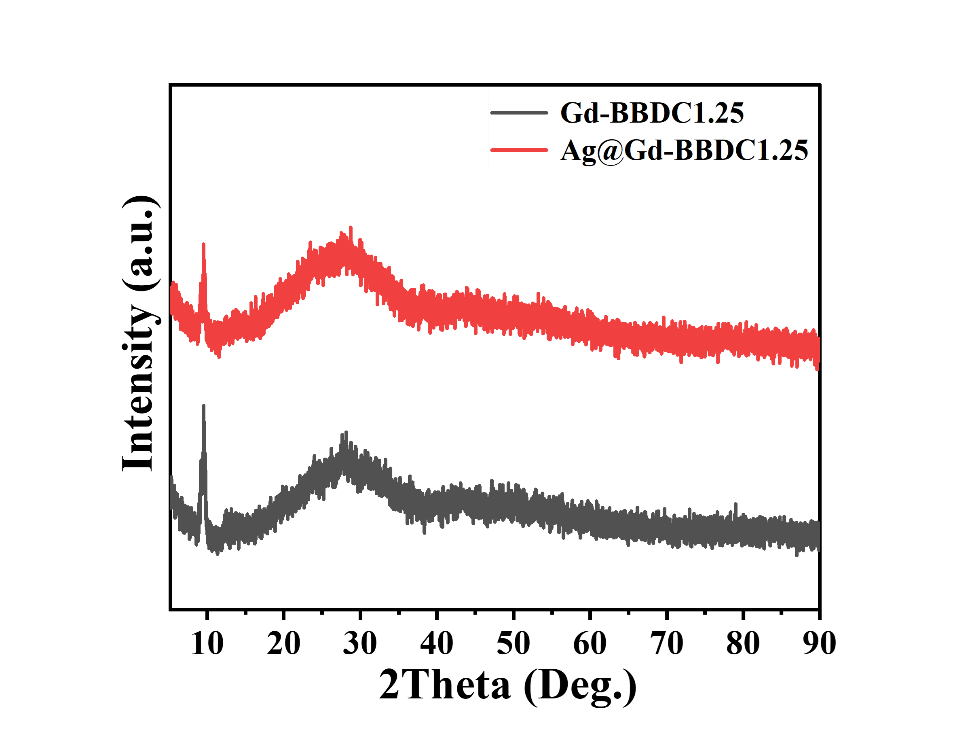


**Figure S13** XRD patterns of Gd-BBDC1.25 and Ag@Gd-BBDC1.25.


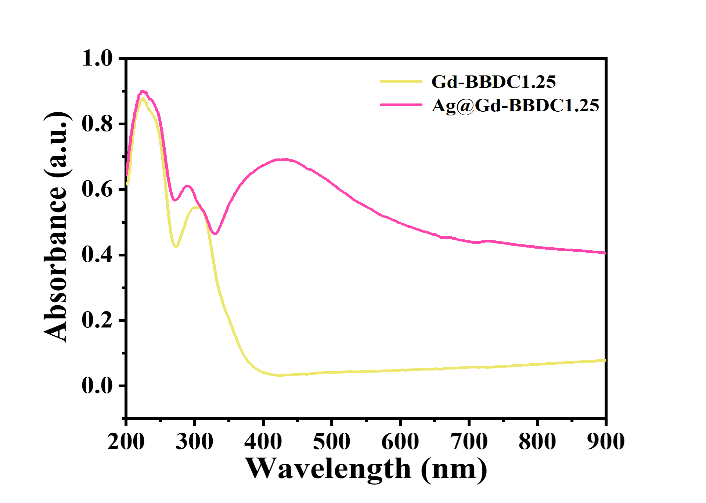


**Figure S14** the UV–visible diffuse reflection spectra (UV-vis DRS) of Gd-BBDC1.25 and Ag@Gd-BBDC1.25.

**
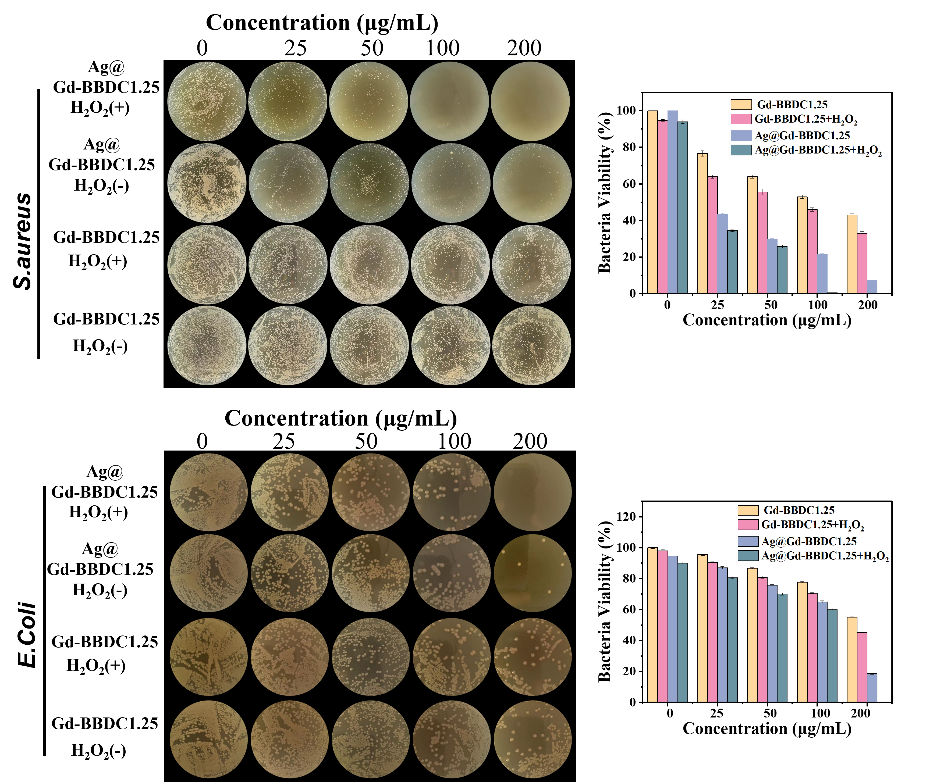
**

**Figure S15** Gd-BBDC1.25 and Ag@Gd-BBDC1.25 solutions with different concentrations were investigated during the antibacterial experiments. In the presence or absence of 1mM H_2_O_2_.

_
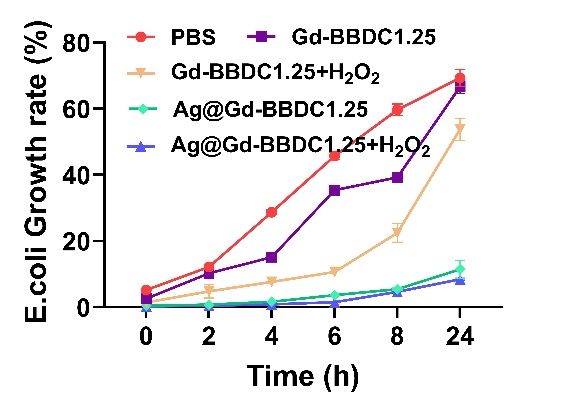
_

**Figure S16** Growth rate of E. coli treated with different groups during 24 h.

**
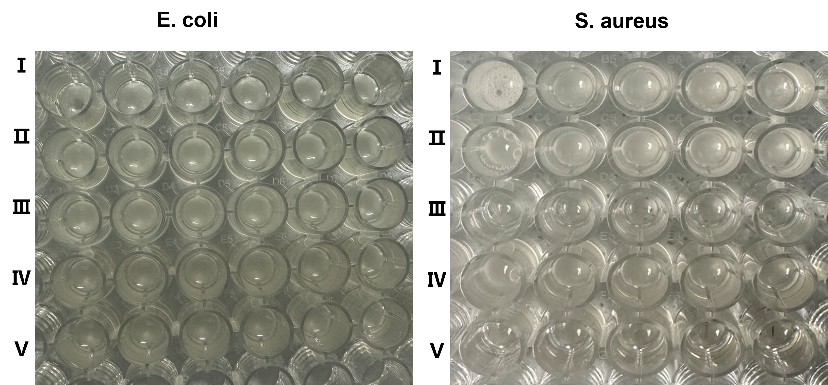
**

**Figure 17** The photographs of bacterial turbidity treated by (Ⅰ) PBS, (Ⅱ) Gd-BBDC1.25, (Ⅲ) Gd-BBDC1.25+H_2_O_2_ (Ⅳ) Ag@Gd-BBDC1.25 (Ⅴ) [Ag@Gd-BBDC1.25+H_2_O_2_](mailto:Ag@Gd-BBDC1.25+H2O2).





**Figure S18** The time-dependent cumulative Ag^+^ release profiles in PBS/H_2_O_2_ solution.


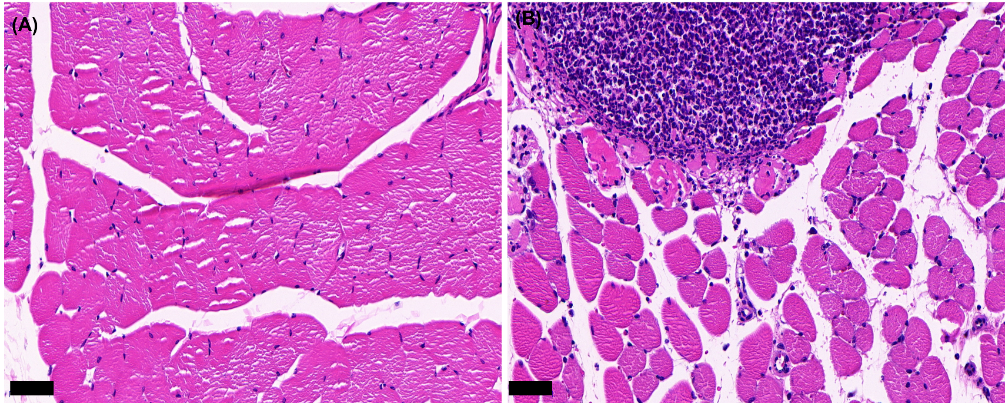


**Figure S 19** Haematoxylin and eosin (H&E) images of thigh muscle of mice after intra-muscularly injected PBS (A), S. aureus (B). scale bar: 50 μm.


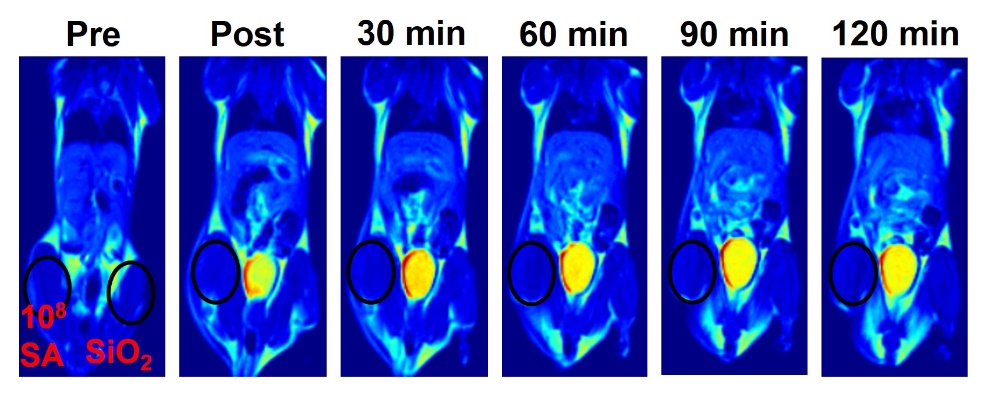


**Figure S20** T_1_-weighted MR images of 10^8^ cfu S. aureus-induced myositis mice in the right thigh muscle after injection of clinical gadolinium-based contrast agent Gd-DTPA.


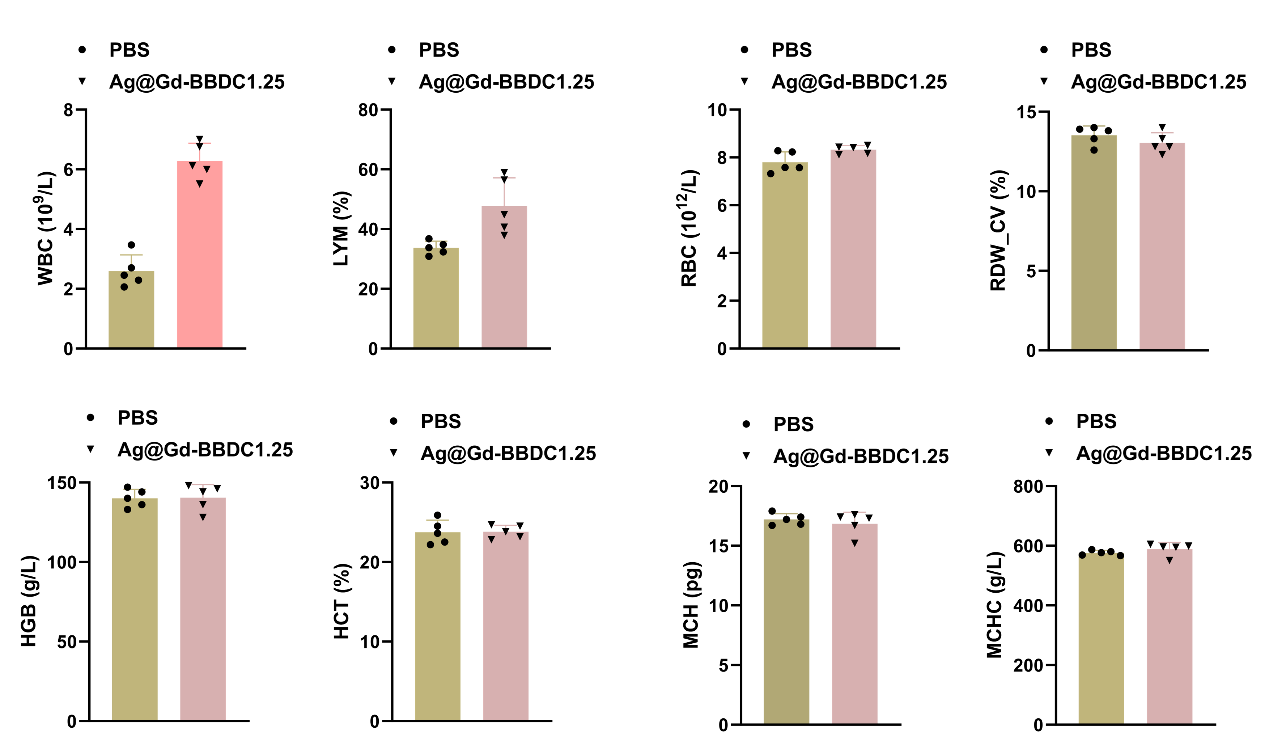


**Figure S21** Hematology parameters of the mice with different treatments (after 24 h treated with saline and Ag@Gd-BBDC1.25) (n = 5, mean ± s.d.).

**
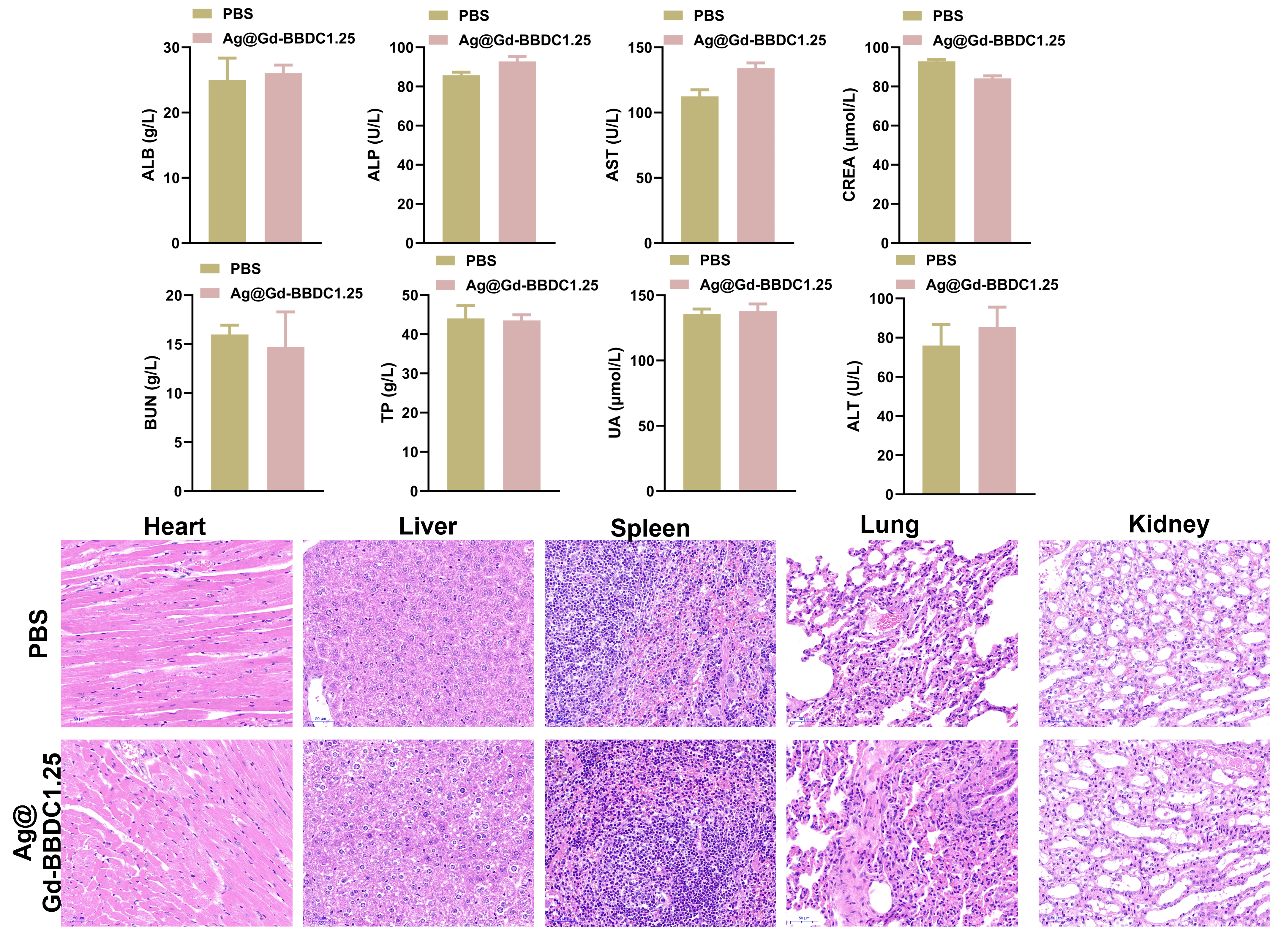
**

**Figure S22** Blood biochemical indexes and pathological H&E-stained images of tissue sections from heart, liver, spleen, lung and kidney of the mice (after 24 h treated with saline and Ag@Gd-BBDC1.25) (n = 5, mean ± s. d.).

References

1. Rieter, W. J.; Taylor, K. M. L.; An, H.; Lin, W.; Lin, W., Nanoscale Metal−Organic Frameworks as Potential Multimodal Contrast Enhancing Agents. *Journal of the American Chemical Society* **2006,** *128* (28), 9024-9025.

2. Qin, L.; Sun, Z.-Y.; Cheng, K.; Liu, S.-W.; Pang, J.-X.; Xia, L.-M.; Chen, W.-H.; Cheng, Z.; Chen, J.-X., Zwitterionic Manganese and Gadolinium Metal–Organic Frameworks as Efficient Contrast Agents for in Vivo Magnetic Resonance Imaging. *ACS Applied Materials & Interfaces* **2017,** *9* (47), 41378-41386.

3. Zhang, H.; Shang, Y.; Li, Y.-H.; Sun, S.-K.; Yin, X.-B., Smart Metal–Organic Framework-Based Nanoplatforms for Imaging-Guided Precise Chemotherapy. *ACS Applied Materials & Interfaces* **2018,** *11* (2), 1886-1895.

4. Tian, C.; Zhu, L.; Lin, F.; Boyes, S. G., Poly(acrylic acid) Bridged Gadolinium Metal–Organic Framework–Gold Nanoparticle Composites as Contrast Agents for Computed Tomography and Magnetic Resonance Bimodal Imaging. *ACS Applied Materials & Interfaces* **2015,** *7* (32), 17765-17775.

5. Wang, G. D.; Chen, H.; Tang, W.; Lee, D.; Xie, J., Gd and Eu Co-Doped Nanoscale Metal–Organic Framework as a T1–T2 Dual-Modal Contrast Agent for Magnetic Resonance Imaging. *Tomography* **2016,** *2* (3), 179-187.

6. Xia, J.; Xue, Y.; Lei, B.; Xu, L.; Sun, M.; Li, N.; Zhao, H.; Wang, M.; Luo, M.; Zhang, C.; Huang, B.; Du, Y.; Yan, C.-H., Multimodal channel cancer chemotherapy by 2D functional gadolinium metal–organic framework. *National Science Review* **2021,** *8* (7).

7. Zhang, S.-Y.; Wang, Z.-Y.; Gao, J.; Wang, K.; Gianolio, E.; Aime, S.; Shi, W.; Zhou, Z.; Cheng, P.; Zaworotko, M. J., A Gadolinium(III) Zeolite-like Metal-Organic-Framework-Based Magnetic Resonance Thermometer. *Chem* **2019,** *5* (6), 1609-1618.
